# Glass crystallization making red phosphor for high-power warm white lighting

**DOI:** 10.1038/s41377-021-00498-6

**Published:** 2021-03-12

**Authors:** Tao Hu, Lixin Ning, Yan Gao, Jianwei Qiao, Enhai Song, Zitao Chen, Yayun Zhou, Jing Wang, Maxim S. Molokeev, Xiaoxing Ke, Zhiguo Xia, Qinyuan Zhang

**Affiliations:** 1 School of Physics and Optoelectronics, State Key Laboratory of Luminescent Materials and Devices and Guangdong Provincial Key Laboratory of Fiber Laser Materials and Applied Techniques, South China University of Technology, Guangzhou, Guangdong China; 2 Anhui Key Laboratory of Optoelectric Materials Science and Technology, Key Laboratory of Functional Molecular Solids, Ministry of Education, Anhui Normal University, Wuhu, Anhui China; 3 School of Applied Physic and Materials, Wuyi University, Jiangmen, Guangdong China; 4 Ministry of Education Key Laboratory of Bioinorganic and Synthetic Chemistry, State Key Laboratory of Optoelectronic Materials and Technologies, KLGHEI of Environment and Energy Chemistry, School of Chemistry and Chemical Engineering, Sun Yat-sen University, Guangzhou, Guangdong China; 5 Laboratory of Crystal Physics, Kirensky Institute of Physics, Federal Research Center KSC SB RAS, Krasnoyarsk, Russia; 6 Siberian Federal University, Krasnoyarsk, Russia; 7 Research and Development Department, Kemerovo State University, Kemerovo, Russia; 8 Institute of Microstructure and Property of Advanced Materials, Beijing University of Technology Beijing, Beijing, China

**Keywords:** Inorganic LEDs, Fluorescence spectroscopy

## Abstract

Rapid development of solid-state lighting technology requires new materials with highly efficient and stable luminescence, and especially relies on blue light pumped red phosphors for improved light quality. Herein, we discovered an unprecedented red-emitting Mg_2_Al_4_Si_5_O_18_:Eu^2+^ composite phosphor (*λ*_ex_ = 450 nm, *λ*_em_ = 620 nm) via the crystallization of MgO–Al_2_O_3_–SiO_2_ aluminosilicate glass. Combined experimental measurement and first-principles calculations verify that Eu^2+^ dopants insert at the vacant channel of Mg_2_Al_4_Si_5_O_18_ crystal with six-fold coordination responsible for the peculiar red emission. Importantly, the resulting phosphor exhibits high internal/external quantum efficiency of 94.5/70.6%, and stable emission against thermal quenching, which reaches industry production. The maximum luminous flux and luminous efficiency of the constructed laser driven red emitting device reaches as high as 274 lm and 54 lm W^−1^, respectively. The combinations of extraordinary optical properties coupled with economically favorable and innovative preparation method indicate, that the Mg_2_Al_4_Si_5_O_18_:Eu^2+^ composite phosphor will provide a significant step towards the development of high-power solid-state lighting.

## Introduction

Solid-state lighting (SSL) has advanced rapidly over the past decades, and will definitely dominate the future lighting market^1–3^. The current standard architecture for SSL is the phosphor-converted light-emitting diode (pc-LED), where the blue LED chip is covered with one or more down-shifting phosphors dispersed in organic binder to produce composite white light^4–7^. Despite those spectacular success in the pc-LED, the notorious “efficiency drop”, that is nonthermal drop in efficiency with increasing input power density^8,9^ precludes pc-LED operation in the fields, where high luminance and luminous fluxes lighting source are required. Recently, laser diode (LD) driven SSL approach, whereby a focused laser beam illuminates a phosphor color converter, can generate luminance far exceeding the state-of-art LED source by factors of 2–10^10^. This way is particularly attractive for automotive headlamp, outdoor lighting, multimedia projectors, laser TVs and so on^11^. However, the thermal shock of laser is extreme, making the traditional organic binders with poor physical and chemical stability undesired for LD applications^12^. Accordingly, extensive efforts are preoccupied in the exploitation of new materials with highly efficient and stable luminescence, including single crystal phosphor, polycrystalline ceramic phosphor, and phosphor-in-glass (PiG)^11,13–16^.

Although various types of bulk phosphors have been designed and constructed successfully so far, actually almost all reports are limited to Ce^3+^-doped garnet-type yellow-emitting PiG/ceramic composite phosphors^17,18^. Apparently, the high-power white lighting device based on “blue laser + yellow-emitting YAG:Ce^3+^ garnet” scheme is still flawed in application for the lack of red component, resulting a pale white light with high correlated color temperature (CCT >7500 K) and low color rendering index (CRI <75)^19,20^. Thus, the discovery of efficient red-emitting bulk phosphor is essential. Concerning this case, particular efforts have been made to fabricate red-emitting CaAlSiN_3_:Eu^2+^ PiG/ceramic composite^21,22^, but few of them can fulfill the high demands generated by practice applications. The reasons are (1) CaAlSiN_3_:Eu^2+^ phosphor unavoidably suffers from erosion when co-sintering it with glass frit at high-temperature^17^, which leads an inferior luminescent performance, viz., a lower quantum efficiency and stronger thermal emission quenching compared with the fresh CaAlSiN_3_:Eu^2+^ phosphor powder. (2) The construction of ceramic is strictly constrained by high-pressure and high-vacuum conditions^15,18,22^, and then such complex and economically unfavorable preparation processes hinder it from industrial production.

Crystallization of inorganic glass helps to realize new crystal formation and transformation for bulk composites with new functionalities in a pressureless, cost-effective, and scalable way in one step^23–25^, as a typical example found in the in situ crystallization of a yellow-emitting Y_3_Al_5_O_12_:Ce^3+^ nano-phosphor from Y_2_O_3_-Al_2_O_3_ glass^25^. Despite that, formidable challenges still remained for this strategy to develop red-emitting Eu^2+^ activate bulk phosphor, and actually there is no report concerning this to the best of our knowledge. The major challenge mainly stems from the fact that one can hardly crystallize a host with suitable magnitude of centroid shift/crystal field splitting energy for the dopant, which can set the energy of 5*d* levels right at red electromagnetic spectrum region. Even so, encouraged by the large degree of freedom in glass composition design and the highly controllable crystallization processes, and thereby it enables us to intentionally manipulate spectroscopic features^26,27^, breathtaking innovations of preparing red-emitting composite phosphor can be achieved prospectively.

Herein, a glass crystallization engineering route for red-emitting phosphor is experimentally demonstrated for the first time. Specifically, by carefully designing the aluminosilicate glass matrix and conducting crystallization, we realize the fabrication of red-emitting Mg_2_Al_4_Si_5_O_18_:Eu^2+^ cordierite phosphor with near-unity photoluminescence quantum efficiency and excellent thermal stability. Noteworthy, cordierite (Mg_2_Al_4_Si_5_O_18_) is a famous material for its technologically important applications in integrated circuit substrates, electronic packaging, automotive catalyst, thermal insulation, and kiln furniture^28^. It is also an excellent candidate for luminescent host accommodating various activators, particularly, by doping Eu^2+^ into Mg_2_Al_4_Si_5_O_18_. Several studies showed Mg_2_Al_4_Si_5_O_18_:Eu^2+^ exhibited single-band blue emission^29–31^, and recently dual-band emission with strong blue and weak red luminescence was also reported^32^. However, it is a challenge to address the Eu^2+^ luminescence and its crystallographic occupancy, and more importantly, one cannot prepare the important red-emitting phosphors pumped by blue light in this system. Herein, novel red Mg_2_Al_4_Si_5_O_18_:Eu^2+^ bulk composite has been reported, and combined experimental and theoretical investigations are performed to uncover the relationships between crystallographic structures and luminescence properties. Moreover, the laser driven red-emitting device constructed using Mg_2_Al_4_Si_5_O_18_:Eu^2+^ exhibits a high luminous flux and luminous efficiency, demonstrating it can serve as efficient color converter for high-power warm white-lighting application. Indeed, a high-quality warm white-lighting with a low CCT (4146 K) and excellent CRI (*R*_a_ = 85.2, *R*_9_ = 64.5) is also fulfilled.

## Results and discussion

### Fabrication of red-emitting Mg_2_Al_4_Si_5_O_18_:Eu^2+^ composite via glass crystallization

**Fig. 1:**
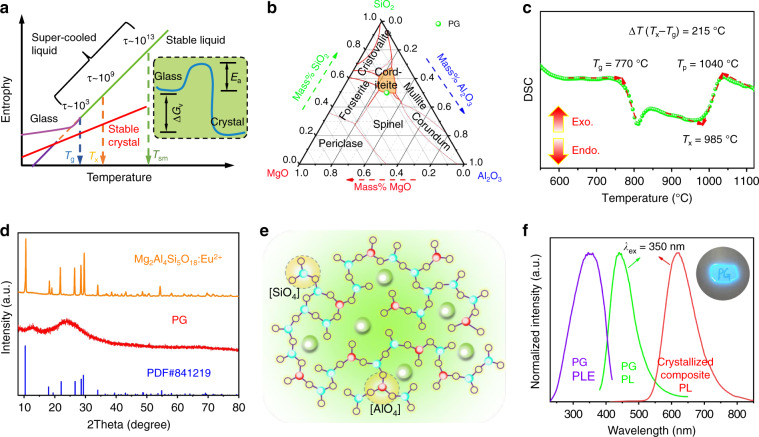
Design and fabrication of Mg_2_Al_4_Si_5_O_18_:Eu^2+^ composite phosphor. **a** Schematic illustration for the entropy of glass, stable crystal, super-cooled liquid, and stable liquid as a function of temperature. *T*_g_, *T*_x_, and *T*_sm_ are the glass transition temperature, crystallization onset temperature, and the melting temperature of stable crystal, respectively. The inset illustrates free energy evolution of the system as glass crystallization proceeds. Δ*G*_v_ means Gibbs free energy difference between metastable glass and stable crystal, and *E*_a_ represents activated energy that needs to be overcome upon glass crystallization. **b** MgO–Al_2_O_3_–SiO_2_ phase diagram, showing the PG composition in wt%. **c** DSC curve of PG recorded at a heating rate of 10 K/min. **d** XRD patterns of PG and the crystallized composite. **e** Schematic illustration of the two-dimensional structure of amorphous aluminosilicate glass network. **f** PL spectra of PG and the crystallized Mg_2_Al_4_Si_5_O_18_:Eu^2+^ composite under 350 nm UV excitation, and PLE of PG measured by monitoring 450 nm emission, the inset photograph shows the PG taken under UV light irradiation

Glass is a thermodynamically metastable (non-equilibrium) solid. After it is heat-treated for a sufficiently long time at higher than glass transition temperature *T*_g_, it will enter into super-cooled state, and then glass structure relaxation could occur, leading to the movement/diffusion of multiple structural building blocks simultaneously or atomic rearrangements orderly. Consequently, precipitating thermodynamically stable (equilibrium) crystal occurred with reducing the free energy of the system, as schematically depicted in Fig. 1a and inset 1a^33^. Theoretically, by carefully selecting glass system and designing chemical compositions (i.e., by controlling over the topological structure of glass network, especially the medium-, or short-range structural arrangement), one may intentionally manipulate nucleation/growth of crystalline within glass as desired. As demonstrated by the typical example of MgO–Al_2_O_3_–SiO_2_ ternary phase diagram^34^ (Fig. 1b), the tailoring of those ternary compositions could selectively crystallize several types of crystallines of spinel, mullite, forsterite, and cordierite, which are all well-known hosts to accommodate optical-active impurities. Herein, aluminosilicate precursor glass (PG) with the nominal chemical composition of 2MgO-Al_2_O_3_−3SiO_2_, which is at the boundary between cordierite (chemical formula of Mg_2_Al_4_Si_5_O_18_) and spinel (Fig. 1b), was specially designed and prepared via melt-quenching method. Broad XRD scattering at about 2*θ* = 25° together without any diffraction peaks confirm the amorphous structure of network of the as-quenched PG. EDS examination shows the homogeneously distribution of elements and its chemical compositions (Fig. S1), with the atom ratio of Mg/Al/Si in accordance with the nominal compositions (Table S1). The thermodynamic parameters of the PG were recorded by DSC thermogram (Fig. 1c), where the glass transition temperature *T*_g_, the onset of crystallization temperature *T*_x_, and maximum crystallization temperature *T*_p_ are determined to be 770, 985, and 1040 °C, respectively. The temperature difference Δ*T* (*T*_x_ − *T*_g_) is calculated to be 215 °C, and such a big value is due to the fact that the glass network structure constructed by Al_2_O_3_ and SiO_2_ is rigid and thermally stable. Specifically, the glass structure relaxation and crystallization were processed via a heat-treatment at 1120 °C, which is slightly higher than *T*_p_. As shown in Fig. 1d, after annealing, the glass hump disappeared completely and the sharp diffraction peaks are indexed to Mg_2_Al_4_Si_5_O_18_ (PDF#841219) phase, manifesting the high crystallinity and the successful preparation of Mg_2_Al_4_Si_5_O_18_ bulk composite. The precipitation of Mg_2_Al_4_Si_5_O_18_ crystal matches well with MgO–Al_2_O_3_–SiO_2_ ternary phase diagram predication (Fig. 1b). Mg_2_Al_4_Si_5_O_18_ is known to crystallize in two polymorphs, high-temperature hexagonal structure and low-temperature orthorhombic. To clarify the structure and the phase purity, Rietveld refinement was conducted with the high-quality synchrotron XRD data by using TOPAS 4.2^35^, as shown in Fig. S2. Almost all peaks were indexed by hexagonal cell (*P*6/*mcc*) with parameters close to Mg_2_Al_4_Si_5_O_18_, except for several weak diffraction peaks of forsterite impurity phase. The amount of Mg_2_Al_4_Si_5_O_18_:Eu^2+^ phase, determined by Rietveld refinement, is 95.8% by weight. Refinement taking hexagonal structure as starting model was stable and gave low *R*-factors (Table S2). Coordinates of atoms and main bond lengths are listed in Tables S3 and S4, respectively.

Due to the sensitivity of Fourier-transform infrared (FTIR) spectra to short-range interactions, the FTIR spectra were measured, aiming to gain knowledge of the network topology evolution from glass to hexagonal Mg_2_Al_4_Si_5_O_18_ crystal. As shown in Fig. S3, the PG exhibits three absorption bands in the region of 400–1400 cm^−1^. The intense bands in 800–1200 cm^−1^ are assigned to the stretching vibrations of the [SiO_4_] tetrahedron with a different number of bridging oxygen atoms (1150 cm^−1^ for Q^4^, 1090 cm^−1^ for Q^3^, and 980 cm^−1^ for Si-O-[NBO, non-bridging oxygen] (Q^3^) per [SiO_4_] tetrahedron). The next intense band between 400 and 600 cm^−1^ are due to bending vibrations of Si–O–Si and Si–O–Al linkages, and the band in the 600–800 cm^−1^ region relates to the stretching vibrations of the Al–O bonds with aluminum ions in four-fold coordination^36^. All those indicate that the glass network essentially consists of [AlO_4_] and [SiO_4_] tetrahedrons (Fig. 1e). The small Mg^2+^ cation acts as network modifier to balance the negative charges of either non-bridging oxygen ions or tetrahedral structure unites. The glass crystallization results in the characteristic modification of FTIR with its profile approximately coinciding with that of the PG, implying that the structure of the PG and crystal in the short-range scale are topologically identical. As a result of the ordered reconstruction of [AlO_4_] and [SiO_4_] tetrahedrons, the absorption peak for the composite becomes split and rather sharp, and the quite intense band at ~575 cm^−1^ is the typical vibration of the 6-membered aluminosilicate rings in Mg_2_Al_4_Si_5_O_18_ crystal^37^. It is believed that heterogeneity structure on the nanometer scale of the PG plays a decisive role in precipitating Mg_2_Al_4_Si_5_O_18_ crystal. The structure and chemical composition of nano-scale heterogeneity part might be closely related to the initial crystalline phase (topological crystalline-like ordering exists)^33^, which favors precipitating Mg_2_Al_4_Si_5_O_18_ when heat energies are being supplied.

In addition to phase transformation engineering, chemical surrounding for the optical-active dopant was tailored simultaneously, resulting in intriguing optical properties. As exhibited in Fig. 1f and inset of Fig. 1f, upon introducing Eu^2+^ dopant into the as-made PG, it exhibits bright blue light at ~450 nm. Interestingly, blue emission is almost vanished and red emission is dominated for the final crystallized composite, suggesting the glass relaxation and crystallization energetically drives the Eu^2+^ dopants from aluminosilicate glass towards the precipitated Mg_2_Al_4_Si_5_O_18_ crystalline lattice with a more prominent crystal filed splitting, and the completely vanishment of blue emission also indicates the high doping efficiency.

### Microstructure analysis

**Fig. 2:**
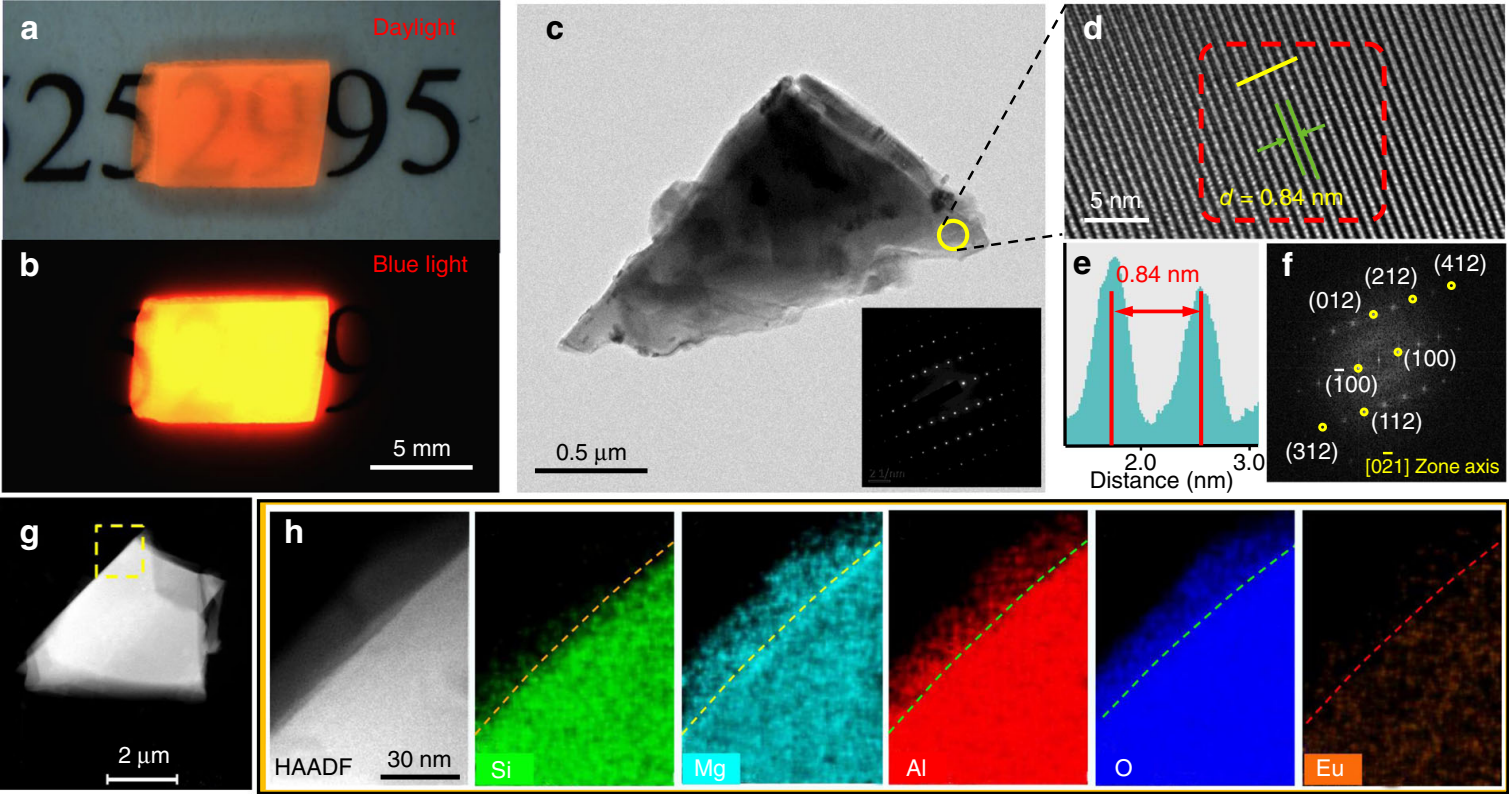
Microstructure of the bulk Mg_2_Al_4_Si_5_O_18_:Eu^2+^ red phosphor. Digital photographs the crystallized composite phosphor with a thickness of 0.4 mm taken under **a** daylight and **b** blue light. **c** Low magnification TEM image of the phosphor particle, the inset is the corresponding selected area electron diffraction (SAED) pattern. **d** High-resolution TEM (HRTEM) pattern. **e** Corresponding intensity profile of **d** across image marked by the dashed green box were obtained. **f** Corresponding FFT image. **g**, **h** HAADF-STEM image and the Energy dispersive X-ray (EDX) mapping of Si, Mg, Al, O, and Eu elements

Figure 2a, b exhibit the photographs of the bulk Mg_2_Al_4_Si_5_O_18_:Eu^2+^ composite phosphor taking under daylight and 450 nm blue light excitation. The apparent body color is orange red vividly, and bright red luminescence is observed upon blue light irradiation (Fig. 2a). It is also at a high level of densification and no pores or voids could be observed at various magnification scales (Fig. S4). TEM image and the corresponding SAED pattern (Fig. 2c and inset of Fig. 2c) demonstrate the single crystalline feature with high crystallinity of microparticle. The high-resolution TEM (HRTEM) pattern shows the distinct lattice fringes (Fig. 2d). A typical interplanar spacing of 0.84 nm is matching well with the (1 0 0) plane of hexagonal Mg_2_Al_4_Si_5_O_18_ crystal, according to the fast Fourier transform (FFT) image viewing along [0-21] zone axis (Fig. 2e, f). There are no lattice fringes at the edge of the crystal (the region surrounded by yellow dotted line in Fig. S5), suggesting it is the tiny remaining glassy phase. High-angle annular dark-field scanning TEM (HAADF-STEM) observations and the corresponding element mappings (Fig. 2g, h) were also carried out to characterize the microstructure. As observed in element mappings, the Mg, Al, Si, and O are homogeneously distributed over the crystal, whereas the Mg, Al, and O are rich over the glassy phase. The absence of Si in the remaining glassy suggests the almost totally depletion of Si in the process of Mg_2_Al_4_Si_5_O_18_ crystallize propagation.

### Site occupancy and optical properties

**Fig. 3:**
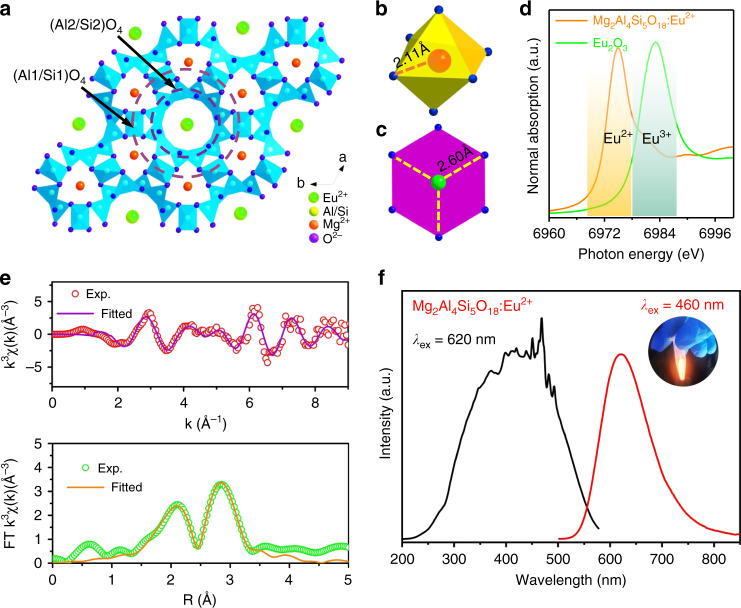
Crystal structure, local structure analysis and optical properties of the Mg_2_Al_4_Si_5_O_18_:Eu^2+^ composite phosphor. **a** 2×2×2 type super-cell of hexagonal Mg_2_Al_4_Si_5_O_18_:Eu^2+^ crystal structure viewing along *c*-axis. The local coordination of **b** MgO_6_ and **c** EuO_6_ polyhedron, showing the Mg–O and Eu–O bond lengths. **d** X-ray absorption near-edge structure (XANES) spectra of the Eu *L*_3_ edge in Mg_2_Al_4_Si_5_O_18_:Eu^2+^ and Eu_2_O_3_. **e** *k*^3^-weighted Eu *L*_3_ edge EXAFS spectra and the corresponding Fourier transform of the Mg_2_Al_4_Si_5_O_18_:Eu^2+^ composite fitting as a function of R. **f** Room temperature PLE and PL spectra of Mg_2_Al_4_Si_5_O_18_:Eu^2+^ composite, the sharp lines around 450 nm in PLE spectra are caused by xenon lamp light scattering and the spectra is uncorrected. The inset shows the powder under 365 nm light UV irradiation

Figure 3a depicts the crystal structure of hexagonal Mg_2_Al_4_Si_5_O_18_:Eu^2+^. The Al and Si atoms are disorderly distributed over two sets of tetrahedral sites to form (Al1/Si1)O_4_ and (Al2/Si2)O_4_ tetrahedrons. The Mg atoms are surrounded by the (Al/Si)_6_O_18_-type 6-membered rings, and are six-fold coordinated to form MgO_6_ octahedrons (Fig. 3b). Comparing the ionic radius of Eu^2+^ (*r*_6-coord._ = 1.17 Å) with that of Mg^2+^ (*r*_6-coord._ = 0.72 Å) suggests that it is unlikely for Eu^2+^ to replace Mg^2+^ because their radius difference is as high as ~62.5%, which is far beyond the limits of the Hume-Rothery rules for atomic substitution^38^.

There are large vacant channels running parallel to the *c*-axis in the Mg_2_Al_4_Si_5_O_18_ (Fig. S6), and it has been reported that the channels have the capability to accommodate a variety of cation ions^39^. Therefore, one can easily speculate that the Eu^2+^ ions inserted at the channel sites. Figure S7a, b demonstrate the two kinds of strikingly different channel sites in the host, namely, *z* = 0.0 or 0.5 site with six-fold coordinated planar oxygen, another is *z* = 0.25 or 0.75 with 12-fold coordinated oxygen^32^. To understand the site occupation, DFT calculations were conducted to evaluate the rel ative occurrence probabilities of Eu^2+^ substitutions at various sites. Before doing so, the occupational disorder at Al1/Si1 (6*f*) and Al2/Si2 (12*l*) sites needs to be modeled first. In accordance with the Rietveld results (Table S3), the occupancy ratios at the two sites were approximated by Al1/Si1 = 5/1 and Al2/Si2 = 1/3. The resultant atomic composition is consistent with the chemical formula. With this approximation, there are totally 1320 different atomic configurations of the unit cell, which when the crystal symmetry is taken into account^40^, reduces to 57 crystallographically independent configurations that are necessary for calculations. The relative occurrence probability (*P*_i_) of each configuration with multiplicity *i* was evaluated with $P_{\rm{i}} = \frac{1}{{Z_{{\rm{tot}}}}}{{\Omega }}_{\rm{i}}{\mathrm{exp}}( - \frac{{E_{\rm{i}}}}{{kT}})$(*i* =1, …, 57), where Z_tot_ is the partition function, *E*_i_ is the relative DFT total energy, *k* is the Boltzmann constant, and *T* is the synthesis temperature of the material. It was found that five distinct configurations dominate the configurational ensemble, with the relative occurrence probabilities *P* = 0.624, 0.132, 0.076, 0.074, and 0.062, respectively. For the other inequivalent configurations, each *P* < 0.006. Based on this, the undoped unit cells with these five configurations were employed for subsequent investigation of Eu^2+^ site occupations.

For Mg_2_Al_4_Si_5_O_18_:Eu^2+^, we have considered two kinds of Eu^2+^ substitutions, that is, isovalent substitution at the host Mg^2+^ site (Eu_Mg_), and occupations within the vacant channel (Eu_vac_) at *z* = 0.0, 0.25, 0.5, and 0.75 with a Mg vacancy (V_Mg_) or two antisite defects (2Al_Si_) as the charge compensator. The DFT calculations revealed that the defect formation energies of Eu_Mg_ and Eu_vac_–V_Mg_ are at least by 2.5 eV higher than the most stable Eu_vac_–2Al_Si_ complexes. This means that Eu_Mg_ and Eu_vac_–V_Mg_ are unlikely to occur compared to Eu_vac_–2Al_Si_, and will thus be discarded in the following discussion. For Eu_vac_ at each site within the vacant channel, there are 45 Al_Si_–Al_Si_ combinations in a given unit cell. As such, for the five most probable atomic configurations of unit cell each with four substituted sites (*z*), a total of 900 Eu^2+^-incorporated different cells have been calculated by DFT to explore the site preference of Eu^2+^ within the vacant channel. The results show that Eu^2+^ locations at *z* = 0.25 and 0.75 sites are unstable in that the Eu^2+^ initially positioned at these sites would relax into *z* = 0.0 or 0.5 sites during DFT geometry optimization. The most stable configurations (to within 100 meV) have Eu^2+^ located at *z* = 0.0 or 0.5 site, surrounded by six O in a quasi-planar structure in the first coordination shell and 2Al + 4Si or 3Al + 3Si atoms randomly distributed in the second coordination shell, which is in line with the occupational disorder of Al/Si sites. Therefore, DFT calculations predict that most Eu^2+^ ions are located at *z* = 0.0 or 0.5 sites with six-fold coordination within the vacant channel (Fig. 3a, c).

To experimentally determine the location of Eu and its valance state, extended X-ray absorption fine structure (EXAFS) at the Eu *L*_3_ edge was measured. Figure 3d shows the Eu *L*_3_ edge X-ray absorption near-edge structure (XANES) spectra of the composite phosphor and Eu_2_O_3_. The absorption energy at about 6975 and 6982 eV are attributed to 2*p*_3/2_ → 5*d* transitions of Eu^2+^ and Eu^3+^, respectively. The absence of 6982 eV peak for the composite suggests that the valence state of Eu in the Mg_2_Al_4_Si_5_O_18_ is in divalent oxidation state, which is also beneficial for achieving high efficiency luminescence. The EXAFS spectra were processed in Athena (version 0.9.25) for background, pre-edge line, and post-edge line calibrations. Then Fourier transformed fitting was carried out in Artemis (version 0.9.25). The *k*^3^ weighting, *k*-range of 3–9 Å^−1^ and *R* range of 1–4 Å were used for the fitting. The four structure parameters, including coordination number (CN), bond length (*R*), Debye–Waller factor (*σ*^2^), and *E*_0_ shift (ΔE_0_) were fitted without anyone was fixed, constrained, or correlated. The reciprocal space *k*^3^ weighting EXAFS and the corresponding Fourier transformed spectra are shown in Fig. 3e. The fitted structure parameters are presented in Table S5. The spectra were well fitted (*R* factor = 0.006), with the fitted CN in the first coordinated shell is 6.5 ± 1.6, and *R* for Eu–O bond is 2.60 ± 0.03 Å. This strongly supports that Eu^2+^ incorporates in the *z* = 0.0 or 0.5 channel sites as the fitted parameters are consistent well with that of the non-doped Mg_2_Al_4_Si_5_O_18_ (the diameter of the channel is ~5.6 Å, and those sites are six-fold coordination), in line with the DFT calculations discussed above. Indeed, cordierite is an incredible phosphor host, and the blue emission in Mg_2_Al_4_Si_5_O_18_:Eu^2+^ was also reported previously, and the origin of the Eu^2+^ occupation is still a controversial issue^29,30^. As such, one can still expect future work to address some important findings on this topic.

Figure 3f displays the photoluminescence excitation and emission spectra of Mg_2_Al_4_Si_5_O_18_:Eu^2+^ composite phosphor measured at room temperature. It exhibits bright red-light emission under near UV light irradiation (inset of Fig. 3f). Under 450 nm blue light excitation, the PL spectrum consists a broad band emission with the maximum peak at ~620 nm, ascribed to parity-allowed electric dipole Eu^2+^: 4*f*^6^5*d* → 4*f*^7^(^8^*S*_7/2_) transition. The excitation spectra by monitoring 620 nm emission exhibits a broad band with the maximum peak at around 450 nm; therefore, the phosphor is blue light highly excitable. Noteworthy, the realization of Eu^2+^ red emission in oxide-based host is known a challenge task. Recently, Stefańska et al. observed the red emission in Mg_2_Al_4_Si_5_O_18_:Eu^2+^ phosphor powder^32^, which was, however, accompanied with the strong blue emission. PL decay of the 4*f*^6^5*d* state displays a nearly single exponent decay (Fig. S8), and time-resolved PL spectra shows an insignificant variation of emission profile at as time prolongs from 7 to 56 μs (Fig. S9), suggesting a homogeneous crystal field environment around Eu^2+^ in Mg_2_Al_4_Si_5_O_18_ lattice. The derived luminescence decay time is 15.4 μs, which is unexpectedly long compared to those (about 1.0 ± 0.5 μs^41^). The origin of this slow decay will be discussed later. Under 450 nm blue light excitation, the composite phosphor exhibits a high internal/external quantum efficiency of 94.5/70.6% (Fig. S10), guaranteeing its practical application.

**Fig. 4:**
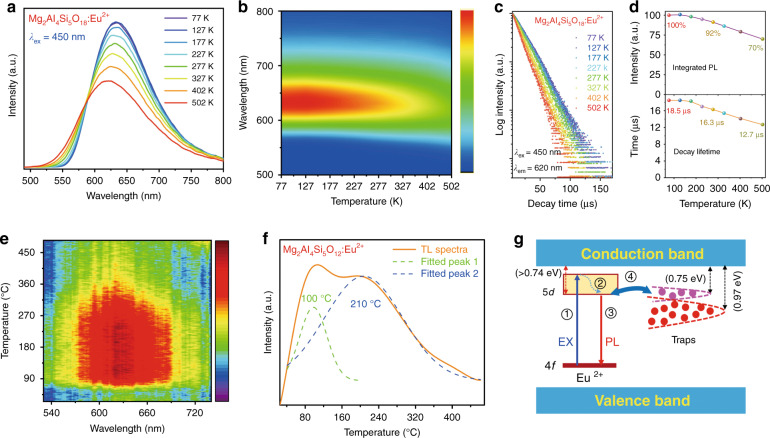
Thermal emission properties of the Mg_2_Al_4_Si_5_O_18_:Eu^2+^ composite phosphor. **a**, **b** Emission spectra of Mg_2_Al_4_Si_5_O_18_:Eu^2+^ (*λ*_ex_ = 450 nm) in the temperature range 77–502 K. **c** Decay curves of Mg_2_Al_4_Si_5_O_18_:Eu^2+^ (*λ*_ex_ = 450 nm, *λ*_em_ = 620 nm) in the temperature range 77–502 K. **d** Normalized integrated PL intensity and decay time as function of temperature. TL glow curves of the Mg_2_Al_4_Si_5_O_18_:Eu^2+^ composite phosphor, **e** three-dimensional TL **f** two-dimensional TL spectra. **g** Schematic illustration of the possible mechanism of the slow decay of Eu^2+^ 5*d* state in Mg_2_Al_4_Si_5_O_18_, showing the processes of electron ① excitation, ② nonradiative relaxation, ③ emission, ④ trapping, and detrapping

As is known, the thermal shock of laser during the application is extreme, with the laser pump power of even a few watts into sub-millimeter spot can easily raise local temperature well into hundreds of degrees Celsius. Therefore, thermally stable properties are necessarily required in laser applications. Temperature-dependent XRD patterns (Fig. S11) verify the phase and physicochemical stability of the as-fabricated Mg_2_Al_4_Si_5_O_18_:Eu^2+^ composite. The emission spectra (*λ*_ex_ = 450 nm) in Fig. 4a, b are gradually blue-shifted and broadened (Fig. S12) with increasing temperature, as commonly observed for Eu^2+^ emission. The blue shift is caused by lattice thermally expansion (Fig. S13), which increases the Eu–O distance and thus results in a smaller crystal field splitting and a decreased covalency. The spectrum broadening is essentially due to the enhanced electron–phonon interaction at high temperature, inducing the electron thermally populate to higher vibrational levels. The PL decay curves at various temperature are close to single-exponential (Fig. 4c). The composite exhibits excellent thermal stable luminescence; the integrated PL intensity at 423 K (150 °C) remains ~78% of that at 70 K (Fig. 4d). The quenching temperature (*T*_0.5_), namely, the temperature at which the emission intensity (or decay time) drops to 50% of the low-temperature value, is higher than 500 K (Fig. 4d), from which the activation energy (*E*_a_) for thermal quenching can be estimated to be larger than 0.74 eV^42^. The thermal conductivity is measured to be ~2.2 W m^−1^ K^−1^, larger than that of the organic binder of ~ 0.1–0.2 W m^−1^ K^[−1 12^ and the co-sintered PiG of ~1 W m^−1^ K^[−1 13^. In addition, thanks to the robust of inorganic aluminosilicate matrices, the phosphor also shows good anti-moisture properties (Fig. S14). All those results indicate the Mg_2_Al_4_Si_5_O_18_:Eu^2+^ phosphor is robust enough to withstand the thermal shock of blue LD and humidity during the applications.

Figure 4e shows the three-dimensional thermoluminescence (TL) spectra of Mg_2_Al_4_Si_5_O_18_:Eu^2+^ composite phosphor. The TL emission peaked at ~620 nm, confirming that the defect-trapped charge carriers were thermally released to generate excited Eu^2+^ giving rise to the red emission. The two-dimensional TL spectrum covers a wide temperature range from 30 to 500 °C (Fig. 4f), indicating a continuous distribution of defect trap depth. The spectrum can be fitted by two Gaussian bands peaking at 100 and 210 °C, for which the trap depths (*E*_T_) can be estimated to be 0.76 and 0.97 eV, respectively, on the basis of the approximate equation *E*_T_ = *T*/500, where the temperature *T* is in units of kelvin. These defects could be oxygen vacancies, which have been commonly observed in oxides synthesized in reducing atmosphere and confirmed to be responsible for thermal behavior of Eu^2+^ luminescence^43^.

As mentioned earlier, the long decay time (15.4 μs) of Eu^2+^-related red emission is striking. Dorenbos has proposed the requirements to be met for Eu^2+^-related anomalous emission, namely, a much larger Stokes shift, a broad emission band, and a longer decay time than the normal Eu^2+^ emission^44^. This anomalous emission originates from the close proximity between the excited Eu^2+^ 5*d* emitting level and the bottom of the host conduction band. Upon Eu^2+^ 4*f* → 5*d* excitation, the electron in the 5*d* level delocalizes into the host band state generating a Eu^3+^-trapped excitonic state, from which the transition to the Eu^2+^ 4*f*^7^ ground state leads to a strongly red-shifted emission compared to the normal 5*d* → 4*f* emission. In the present case of Mg_2_Al_4_Si_5_O_18_:Eu^2+^, however, the red emission is considered to be normal because (1) the Stokes shift (3700 cm^−1^) is not large, (2) the emission band (FWHM = 2700 cm^−1^) is not broad, and (3) the emitting level is well below the host conduction band (*E*_a_ > 0.74 eV). If so, what is the cause for the surprising long-decay of the red 5*d* → 4*f* emission?

In Mg_2_Al_4_Si_5_O_18_:Eu^2+^, the TL measurement revealed a distribution of defect trap levels with main peaks at 0.76 and 0.97 eV below the host conduction band edge (Fig. 4g). The energy positions of these defect levels are close to that of Eu^2+^ 5*d* emitting level (>0.74 eV below the host conduction band edge). At room temperature, the electrons trapped at these defect levels can be thermally activated to release into Eu^2+^ 5*d* levels, which results in 5*d* → 4*f* emission with a long decay time. At lower temperature the luminescence decay would be longer in view of the thermally activated electron transfer, consistent with experimental observations (Fig. 4c). A schematic representation of the luminescence mechanism is depicted in Fig. 4g. Upon 450 nm irradiation, Eu^2+^ is excited from the 4*f* ground state to higher 5*d* levels (process 1), which then relaxes nonradiatively to the lowest 5*d* level (process 2). From this excited level, the 5*d* electron can return back to the 4*f* ground state by emitting a red photon (process 3), or transfer to nearby electron-trapping defects by direct tunneling (process 4) due the close proximity between the Eu^2+^ 5*d* and defect levels. During the decay-time measurement of the 5*d* → 4*f* emission, the electron trapped at the defects will transfer back (process 4) to the emitting 5d level, leading to luminescence decay time much longer than the normal case.

### Demonstration of application for LD lighting

**Fig. 5:**
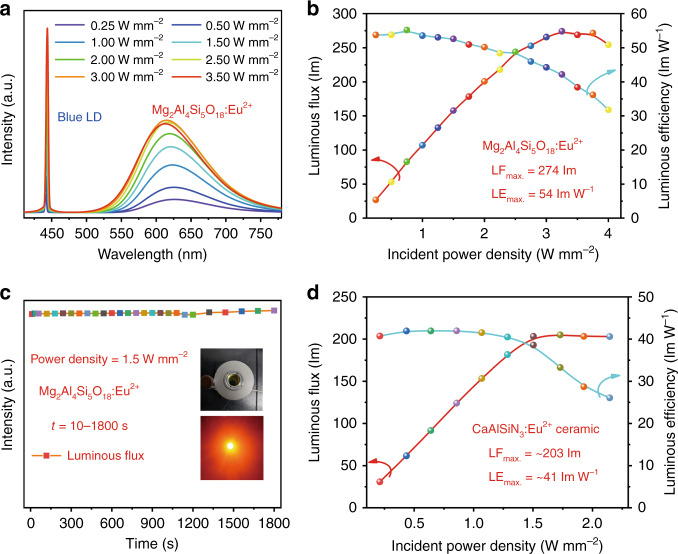
Photoelectric properties of the LD driven red-emitting device constructed by using Mg_2_Al_4_Si_5_O_18_:Eu^2+^ phosphor. The power density-dependent **a** luminescent spectra **b** luminous flux and luminous efficiency of Mg_2_Al_4_Si_5_O_18_:Eu^2+^ composite under the excitation of 445 nm blue laser. **c** Time-dependent luminous flux of the composite at a fixed incident power density of 1.5 W mm^−2^, the inset show the photograph of red LD device. **d** The incident power density-dependent luminous flux and luminous efficiency of the CaAlSiN_3_:Eu^2+^ ceramic^22^

The blue laser driven red lighting device was constructed by coupling the Mg_2_Al_4_Si_5_O_18_:Eu^2+^ composite with 445 nm blue laser diode aiming to evaluate its potential application for high power lighting. As plots in Fig. 5a, b, the emission intensity of the composite increases monotonously as the laser power density increases from 0.25 to 3.25 W mm^−2^, beyond which luminescence saturation occurs. The maximum luminous flux and luminous efficiency achieved for Mg_2_Al_4_Si_5_O_18_:Eu^2+^ composite phosphor is ~274 lm and 54 lm W^−1^, respectively. The time-dependent emission spectra under a fixed power density of 1.5 W mm^−2^ (Fig. S15), and the corresponding luminous flux (Fig. 5c) demonstrate the composite phosphor is stable enough in practice application. We also made a performance comparison between our composite and the previously reported red CaAlSiN_3_:Eu^2+^ ceramic synthesized by using the state-of-art red CaAlSiN_3_:Eu^2+^ phosphor. As shown in Fig. 5d, the maximum luminous flux and luminous efficiency achieved for CaAlSiN_3_:Eu^2+^ ceramic is about 203 lm and 4 l lm W^[−1 22^, which is inferior to our reported photoelectric properties and actually the photoelectric properties realized in Mg_2_Al_4_Si_5_O_18_:Eu^2+^ composite is almost the highest rank among red bulk phosphors. Anyway, all those results suggest that the Mg_2_Al_4_Si_5_O_18_:Eu^2+^ holds potential for addressing the lack of commercially available all-inorganic red-emitting bulk color converter.

**Fig. 6:**
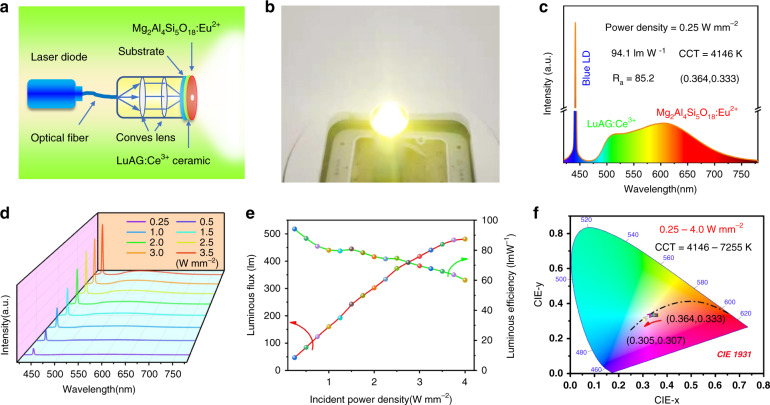
Photoelectric properties of the LD driven white lighting device. **a** Schematic illustration of the construction of blue laser driven white light device. **b** The photograph of the constructed laser driven white light taken under its operation. **c** The emission spectra of the constructed laser driven light device under the incident power density of 0.25 W mm^−2^. Incident power density-dependent **d** emission spectra, **e** luminous flux and luminous efficiency, **f** CIE coordinates of the laser driven lighting device

Finally, we constructed the blue laser driven white lighting device by using a stack package configuration, in which the commercial LuAG:Ce^3+^ ceramic serving as a green color converter is stack packed with red Mg_2_Al_4_Si_5_O_18_:Eu^2+^ to form white light (Fig. 6a, b). Figure 6c demonstrates the emission spectra of the white lighting device operating under the incident power density of 0.25 W mm^−2^. The device exhibits a low correlated color temperature (CCT) of 4146 K, high color render index (*R*_a_ = 85.2, *R*_9_ = 64.5), and CIE chromaticity coordinates of (0.364, 0.333), which is much better than the properties of the YAG:Ce^3+^-based laser driven white lighting featuring a high CCT (>7500 K) and a low *R*_*a*_ (<75)^15^. The incident power density dependent emission spectra, luminous flux and luminous efficiency are presented in Fig. 6d, e and Table S6. With the increment of pumping power density from 0.25 to 4 W mm^−2^, the output of the white light is get enhanced gradually, and the CIE chromaticity coordinates at various power densities are near at Planckian locus curve (Fig. 6f). The maximum luminous flux (480 lm) and luminous efficiency (94.1 lm W^−1^) realized in our white lighting device is relatively low compared with that of the YAG:Ce^3+^-based laser driven white lighting device, partly due to the output of green light is blocked by the red composite. We think there is a large space to improve it via carefully design packing configuration or material design, for example, by further optimization of the transparency of the red composite, or even fabrication of LuAG:Ce^3+^/Mg_2_Al_4_Si_5_O_18_:Eu^2+^ binary composite.

## Conclusions

In summary, we report a facile elaboration of new type of red-emitting Mg_2_Al_4_Si_5_O_18_:Eu^2+^ composite phosphor based on glass relaxation and crystallization of a composition specially designed aluminosilicate glass at ambient conditions. The optical properties realized here are especially surprising, because red phosphor exhibits near unity luminescence efficiency and stable emission against temperature, which enables it readily meets the high-power laser driven lighting applications. Our demonstration represents the first proof-of-principle glass crystallization of red-emitting composite phosphor, and we believe that it will provide a great step toward the advancement of new materials discovery of the solid-state lighting technology for the new photonic applications.

## Methods

### Materials and preparation

The precursor glass (PG) with nominal composition of 2MgO–Al_2_O_3_–3SiO_2_ was prepared via melt-quenching method, and a small amount of Eu_2_O_3_ was introduced into the PG to serve as the source of Eu^2+^ dopant. In a detailed preparation procedure, the raw materials of MgO (1.1612 g), Al_2_O_3_ (2.039 g), SiO_2_ (3.605 g), and Eu_2_O_3_ (optimal doping amount of 0.05 g) were weighted, mixed, and ground thoroughly in an agate mortar. The mixture was transferred into corundum crucible, and then fully melted at 1550 °C for 4 h under a reducing atmosphere (N_2_/H_2_ = 80%/20%) in the tube furnace. The melt was cooled naturally to 750 °C in the tube furnace to form PG and further annealed at 750 °C for 5 h to relinquish the internal stress. The red-emitting Mg_2_Al_4_Si_5_O_18_:Eu^2+^ bulk composite was prepared via heat treatment of the PG at 1120 °C for 15 min under a reducing atmosphere (N_2_/H_2_ = 80%/20%). However, blue-emitting Mg_2_Al_4_Si_5_O_18_:Eu^2+^ bulk composite can be also prepared by using the same method, while with the different PG composition, 2MgO–Al_2_O_3_–4SiO_2_. The obtained PG and composite phosphor were cut and polished before characterizations.

Worthy to mention, the glass melt liquid usually needs to be cooled down at a sufficiently high rate to escape crystallization, so that the liquid melt should be taken out from furnace at high temperature and quenched as fast as possible, which poses extra-cost and dangerous. Commendably, the PG in our case can be fabricated in a more convenient way of melt slowly cooled in the furnace. This added advantage is benefited from a relatively high viscosity (*τ*) of silicate melt and a steep increase of viscosity with decreasing temperature (Fig. 1a), leading to large-scale atomic rearrangements are no longer possible.

### Characterization

The thermodynamic parameters of the PG were measured by a DTA method on the NETZSCH STA 449C with the heating rate of 10 K min^−1^. The thermal conductivity was measured on Physical Property Measurement System (ppms-9). The phase identifications were performed by using powder X-ray diffraction, on Aeris X-ray diffractometer operating at 40 KV and 15 mA. Synchrotron XRD pattern for Rietveld analysis was collected at high-resolution powder diffraction end station (*λ* = 0.82656 Å, 15 KeV) and MYTHEN 24 K detector. The Eu valance state was examined by Eu *L*_3_-edge of X-ray absorption near-edge structure spectroscopy in fluorescence mode. The Rietveld refinement was performed by using TOPAS 4.2 software. The SEM image and elemental mapping of the PG were acquired on HITACHI Regulus 8100 scanning electron microscope equipped with an energy-dispersive X-ray spectroscopy (EDS) system. Scanning transmission electron microscopy (STEM) image were taken on a FEI aberration-corrected Titan Cubed S-Twin transmission electron microscope operating in a high-angle annular dark-field (HAADF) mode equipped with an energy dispersive X-ray spectroscope operated at 200 KV. High-resolution transmission electron microscopy (HRTEM) image and selected electron diffraction (SAED) were taken on transmission electron microscope (JEOL, JEM-2100 and JEM-2010) operating at 200 KV. A Bruker vector 33 spectrometer is used for the measurement of Fourier-transform infrared (FTIR) spectra. The PL and PLE spectra, quantum efficiency, temperature-dependent PL, and temperature-dependent fluorescent decay curves were all recorded on an Edinburgh Instrument FLS1000 spectrofluorometer equipped with a xenon lamp (450 W) as the excitation source. The photoelectric properties of the red emitting device fabricated by Mg_2_Al_4_Si_5_O_18_:Eu^2+^ composite under the high-power 445 nm blue laser excitation, and the white lighting device fabricated by coupling commercial green-emitting LuAG:Ce^3+^ ceramic (purchased from Suzhou Chuangside New Material Co. ltd) and the Mg_2_Al_4_Si_5_O_18_:Eu^2+^ composite under the high-power 445 nm blue laser excitation were measured using an integrating sphere of 1.0 m in diameter connected to a CCD detector.

### Computational methodology

Periodic density functional theory (DFT) calculations on Mg_2_Al_4_Si_5_O_18_:Eu^2+^ were conducted using the Perdew-Burke-Ernzerhof (PBE)^45^ functional and its PBE + U variant with *U*_eff_ = 2.5 eV for the Eu 4*f* electrons^46,47^, as implemented in the Vienna Ab initio Simulation Package (VASP) code^48,49^. Mg(2p^6^3s^2^), Al(3s^2^3p^1^), Si(3s^2^3p^6^), O(2s^2^2p^4^), and Eu(5s^2^5p^6^4f^7^6s^2^) were treated as valence electrons, and their interactions with the respective cores were described by the projected augmented wave approach^50^. The investigation of Eu^2+^-doping in Mg_2_Al_4_Si_5_O_18_ was modeled by incorporating a Eu in the unit cell (58 atoms), with appropriate charge compensators as described in the text. The atomic structures of undoped and doped supercells were fully optimized until the total energies and the forces on the atoms converged to 10^−6^ eV and 0.01 eV Å^−1^. A 2 × 2 × 2 k-point grid was used, and the cutoff energy for the plane wave basis was set to 530 eV. The formation energies of Eu^2+^-related isolated defects or defect complexes were calculated using Δ*E*_f_ = *E*(doped) − *E*(undoped) + $\mathop {\sum }\limits_{\rm{A}} \Delta n_{\rm{A}}\mu _{\rm{A}}$, where *E*(doped) and *E*(undoped) denote DFT total energies of the doped and undoped unit cells, respectively. Δ*n*_A_ is the number of species *A* (=Eu, Mg, Al, or Si) removed from the undoped cell to introduce point defects, and *μ*_A_ is the corresponding atomic chemical potential, which was approximated by the energy of the corresponding metallic atom in view of the fact that the material was prepared in the reducing atmosphere.

## Supplementary information

SUPPLEMENTARY INFORMATION for Glass crystallization making red phosphor for high-power warm white lighting

## References

[1] Pust P, Schmidt PJ, Schnick W (2015). A revolution in lighting. Nat. Mater..

[2] Pimputkar S (2009). Prospects for LED lighting. Nat. Photonics.

[3] Schubert EF, Kim JK (2005). Solid-state light sources getting smart. Science.

[4] Xia ZG, Liu QL (2016). Progress in discovery and structural design of color conversion phosphors for LEDs. Prog. Mater. Sci..

[5] Kim YH (2017). A zero-thermal-quenching phosphor. Nat. Mater..

[6] Pust P (2014). Narrow-band red-emitting Sr[LiAl_3_N_4_]: Eu^2+^ as a next-generation LED-phosphor material. Nat. Mater..

[7] Zhao M (2018). Next-generation narrow-band green-emitting RbLi(Li_3_SiO_4_)_2_:Eu^2+^ phosphor for backlight display application. Adv. Mater..

[8] Wierer JJ, Tsao JY, Sizov DS (2013). Comparison between blue lasers and light-emitting diodes for future solid-state lighting. Laser Photonics Rev..

[9] Cho J, Schubert EF, Kim JK (2013). Efficiency droop in light-emitting diodes: challenges and countermeasures. Laser Photonics Rev..

[10] Lenef A (2019). Phosphor performance under high intensity excitation by InGaN laser diodes. ECS J. Solid State Sci. Technol..

[11] Li SX (2018). Color conversion materials for high-brightness laser-driven solid-state lighting. Laser Photonics Rev..

[12] Huang JL (2015). Rapid degradation of mid-power white-light LEDs in saturated moisture conditions. IEEE Trans. Device Mater. Reliab..

[13] Lin H (2018). Glass ceramic phosphors: towards long-lifetime high-power white light-emitting-diode applications-a review. Laser Photonics Rev..

[14] Arjoca S (2015). Temperature dependence of Ce:YAG single-crystal phosphors for high-brightness white LEDs/LDs. Mater. Res. Express.

[15] Yao Q (2020). YAG:Ce^3**+**^ transparent ceramic phosphors brighten the next-generation laser-driven lighting. Adv. Mater..

[16] Zhang R (2014). A new-generation color converter for high-power white LED: transparent Ce^3+^:YAG phosphor-in-glass. Laser Photonics Rev..

[17] Zhang D (2020). Highly efficient phosphor-glass composites by pressureless sintering. Nat. Commun..

[18] Huang P (2020). Nano wave plates structuring and index matching in transparent hydroxyapatite-YAG: Ce composite ceramics for high luminous efficiency white light-emitting diodes. Adv. Mater..

[19] Hoerder GJ (2019). Sr[Li_2_Al_2_O_2_N_2_]:Eu^2+^-A high performance red phosphor to brighten the future. Nat. Commun..

[20] Qiao JW (2019). Site-selective occupancy of Eu^2+^ toward blue-light-excited red emission in a Rb_3_YSi_2_O_7_:Eu phosphor. Angew. Chem. Int. Ed..

[21] Wang R (2020). Red-emitting improvement of CaAlSiN_3_:Eu^2+^ phosphor-in-glass: insight into the effect of atmospheric pressure preparation on photoluminescence properties and thermal degradation. J. Lumin..

[22] Li SX (2017). New insights into the microstructure of translucent CaAlSiN_3_:Eu^2+^ phosphor ceramics for solid-state laser lighting. J. Mater. Chem. C.

[23] Allix M (2012). Highly transparent BaAl_4_O_7_ polycrystalline ceramic obtained by full crystallization from glass. Adv. Mater..

[24] Alahraché S (2011). Crystallization of Y_2_O_3_-Al_2_O_3_ rich glasses: synthesis of YAG glass-ceramics. J. Phys. Chem. C..

[25] Ma XG (2018). Pressureless glass crystallization of transparent yttrium aluminum garnet-based nanoceramics. Nat. Commun..

[26] Zhou SF (2010). Simultaneous tailoring of phase evolution and dopant distribution in the glassy phase for controllable luminescence. J. Am. Chem. Soc..

[27] Chen DQ (2019). Simultaneous tailoring of dual-phase fluoride precipitation and dopant distribution in glass to control upconverting luminescence. ACS Appl. Mater. Interfaces.

[28] Chowdhury A (2007). Synthesis, properties and applications of cordierite ceramics, Part 1. Int. Ceram. Rev..

[29] Chen J (2014). The luminescence properties of novel α-Mg_2_Al_4_Si_5_O_18_:Eu^2+^ phosphor prepared in air. RSC Adv..

[30] Zhou J (2015). New insight into phase formation of M_*x*_Mg_2_Al_4 + *x*_Si_5 - *x*_O_18_:Eu^2+^ solid solution phosphors and its luminescence properties. Sci. Rep..

[31] Song K (2020). Synthesis and luminescence characteristics of Mg_2_Al_4_Si_5_O_18_:Eu^2+^ and nitrided Mg_2_Al_4_Si_5_O_18_:Eu^2+^ phosphors. J. Lumin..

[32] Stefańska D, Dereń PJ (2020). High efficiency emission of Eu^2+^ located in channel and Mg-site of Mg_2_Al_4_Si_5_O_18_ cordierite and its potential as a Bi-functional phosphor toward optical thermometer and white LED application. Adv. Optical Mater..

[33] Komatsu T (2015). Design and control of crystallization in oxide glasses. J. Non-Cryst. Solids.

[34] Höland, W. & Beall, G. H. *Glass-Ceramic Technology* 2nd edn (Wiley, 2012).

[35] Bruker, A. X. S. *TOPAS, V4: General Profile and Structure Analysis Software for Powder Diffraction Data-User’s Manual* (Bruker AXS, 2008).

[36] Clayden NJ (1999). Solid state ^27^Al NMR and FTIR study of lanthanum aluminosilicate glasses. J. Non-Cryst. Solids.

[37] Mozgawa W, Sitarz M (2002). Vibrational spectra of aluminosilicate ring structures. J. Mol. Struct..

[38] Hume-Rothery W, Powell HM (1935). On the theory of super-lattice structures in alloys. Z. f.ür. Kristallographie.

[39] Thomas P (1991). Powder neutron diffraction study of alkali-substituted cordierites with M_*x*_Mg_2_A1_4+*x*_Si_5-*x*_O_18_ (M = K, Cs; 0 < x ≤ 1) formula. Eur. J. Solid State Inorg. Chem..

[40] Grau-Crespo R (2007). Symmetry-adapted configurational modelling of fractional site occupancy in solids. J. Phys. Condens. Matter.

[41] Poort SHM, Meyerink A, Blasse G (1997). Lifetime measurements in Eu^2+^-doped host lattices. J. Phys. Chem. Solids.

[42] Dorenbos P (2005). Thermal quenching of Eu^2+^ 5d-4f luminescence in inorganic compounds. J. Phys. Condens. Matter.

[43] Qiao JW (2018). Eu^2+^ site preferences in the mixed cation K_2_BaCa(PO_4_)_2_ and thermally stable luminescence. J. Am. Chem. Soc..

[44] Dorenbos P (2003). Anomalous luminescence of Eu^2+^ and Yb^2+^ in inorganic compounds. J. Phys. Condens. Matter.

[45] Perdew JP, Burke K, Ernzerhof M (1996). Generalized gradient approximation made simple. Phys. Rev. Lett..

[46] Canning A (2011). First-principles study of luminescence in Ce-doped inorganic scintillators. Phys. Rev. B.

[47] Dudarev SL (1998). Electron-energy-loss spectra and the structural stability of nickel oxide: an LSDA + U study. Phys. Rev. B.

[48] Kresse G, Furthmüller J (1996). Efficient iterative schemes for ab initio total-energy calculations using a plane-wave basis set. Phys. Rev. B.

[49] Kresse G, Joubert D (1999). From ultrasoft pseudopotentials to the projector augmented-wave method. Phys. Rev. B.

[50] Blöchl PE (1994). Projector augmented-wave method. Phys. Rev. B.

