# Supplementary material for: Glass crystallization making red phosphor for high-power warm white lighting
Source: Light Sci Appl. 2021 Mar 12;10:56. doi: 10.1038/s41377-021-00498-6 (PMC7955133; doi:10.1038/s41377-021-00498-6)
Supplement: Supplementary file 1 — SUPPLEMENTARY INFORMATION for Glass crystallization making red phosphor for high-power warm white lighting [file 41377_2021_498_MOESM1_ESM.docx]

**Supplementary Information**

**Glass crystallization making red phosphor for high-power warm white lighting**

Tao Hu^1^, Lixin Ning^2,*^, Yan Gao^3^, Jianwei Qiao^1^, Enhai Song^1^, Zitao Chen^1^, Yayun Zhou^1^, Jing Wang^4^, Maxim S. Molokeev^5,6,7^, Xiaoxing Ke^8^, Zhiguo Xia^1,*^ Qinyuan Zhang^1,*^

^1^School of Physics and Optoelectronics, State Key Laboratory of Luminescent Materials and Devices and Guangdong Provincial Key Laboratory of Fiber Laser Materials and Applied Techniques, South China University of Technology, Guangzhou, Guangdong 510641, China

^2^Anhui Key Laboratory of Optoelectric Materials Science and Technology, Key Laboratory of Functional Molecular Solids, Ministry of Education, Anhui Normal University, Wuhu, Anhui 241000, China

^3^School of Applied Physic and Materials, Wuyi University, Jiangmen, Guangdong 529020, China

^4^Ministry of Education Key Laboratory of Bioinorganic and Synthetic Chemistry, State Key Laboratory of Optoelectronic Materials and Technologies, KLGHEI of Environment and Energy Chemistry, School of Chemistry and Chemical Engineering, Sun Yat-sen University, Guangzhou, Guangdong 510275, China

^5^Laboratory of Crystal Physics, Kirensky Institute of Physics, Federal Research Center KSC SB RAS, Krasnoyarsk 660036, Russia

^6^Siberian Federal University, Krasnoyarsk 660041, Russia

^7^Research and Development Department, Kemerovo State University, Kemerovo, 650000, Russia

^8^Institute of Microstructure and Property of Advanced Materials, Beijing University of Technology Beijing, Beijing 100124, China

Corresponding Author

* E-mail: ninglx@mail.ahnu.edu.cn, xiazg@scut.edu.cn, qyzhang@scut.edu.cn

**
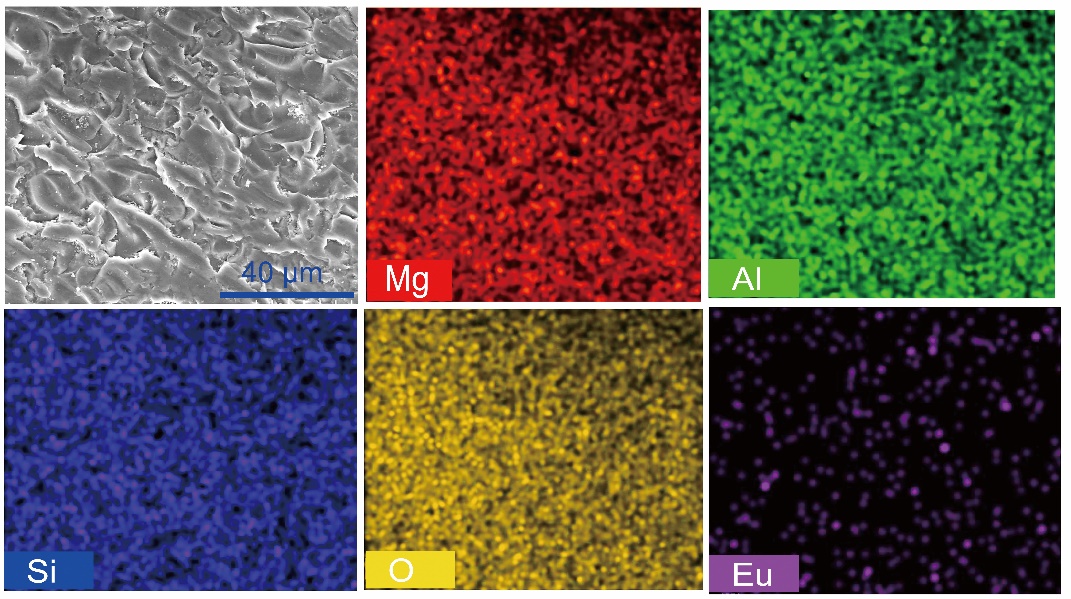
**

**Fig. S1:** SEM image of the PG and the elements (Mg, Al, Si, O, and Eu) mapping.

**
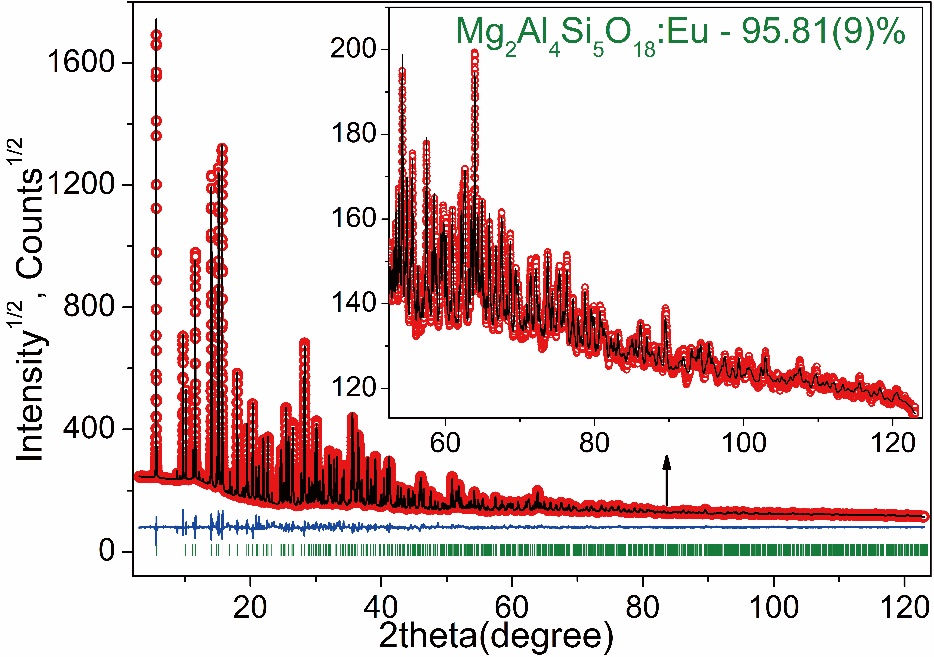
**

**Fig. S2:** Synchrotron radiation XRD profile for Rietveld refinement result of the crystallized composite.


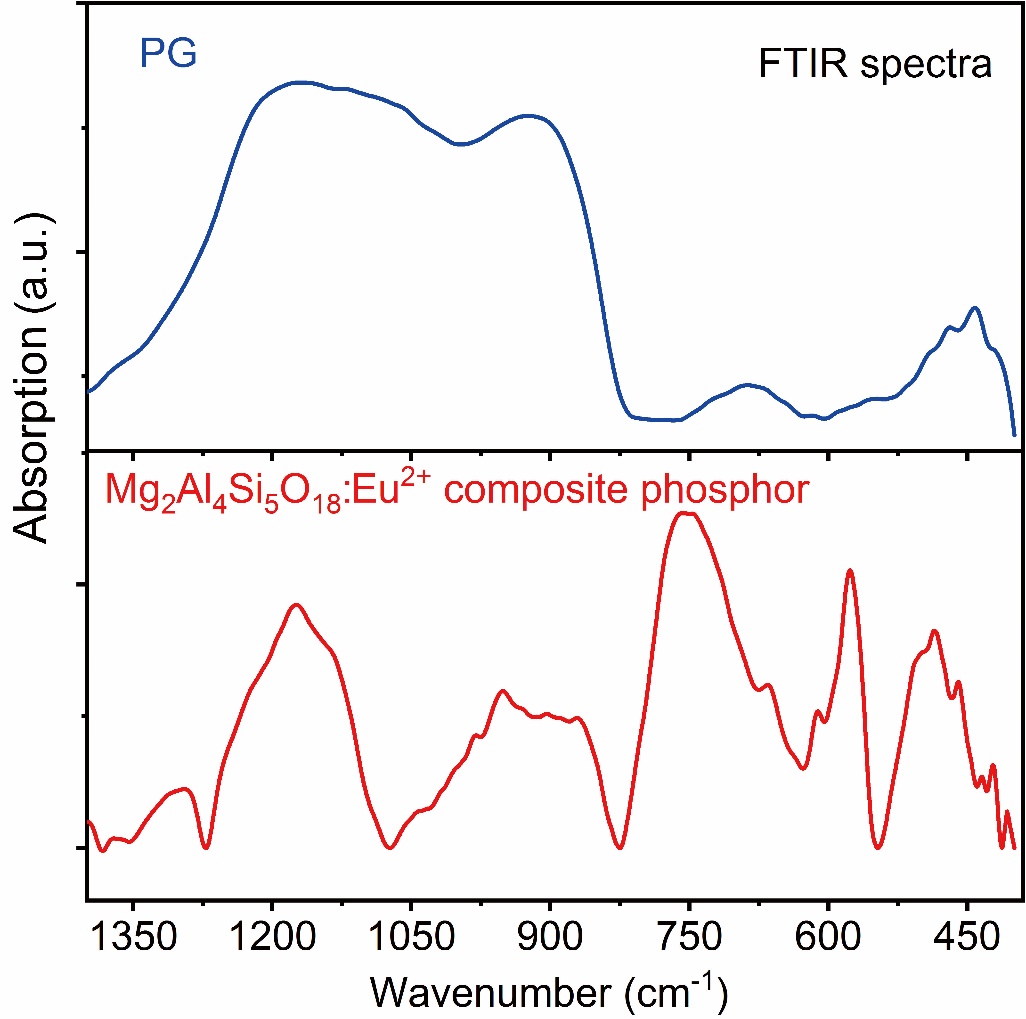


**Fig. S3:** Fourier-transform infrared (FTIR) spectra of the PG and the crystallized composite.


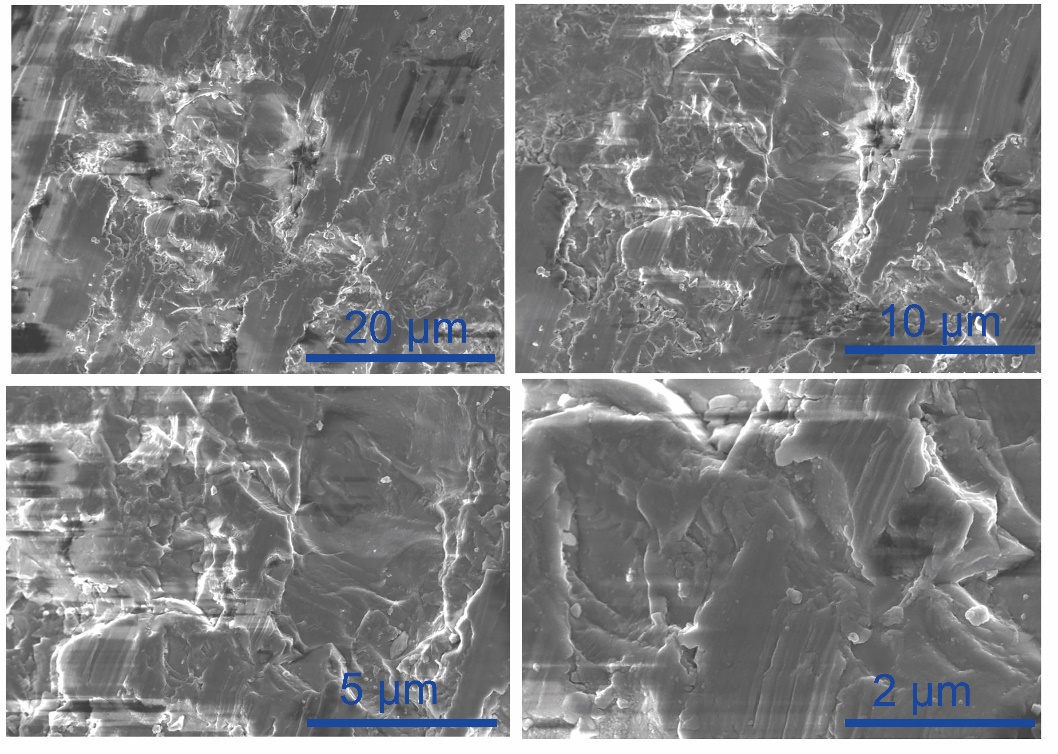


**Fig. S4:** SEM images of the crystallized composite taken under various magnification scales.


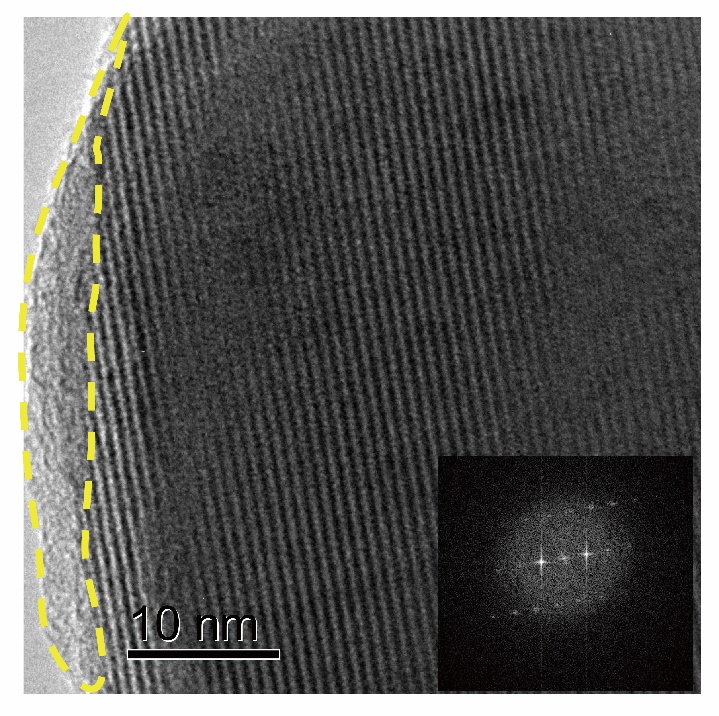


**Fig. S5:** HRTEM pattern of the particle, the inset is the FFT image.


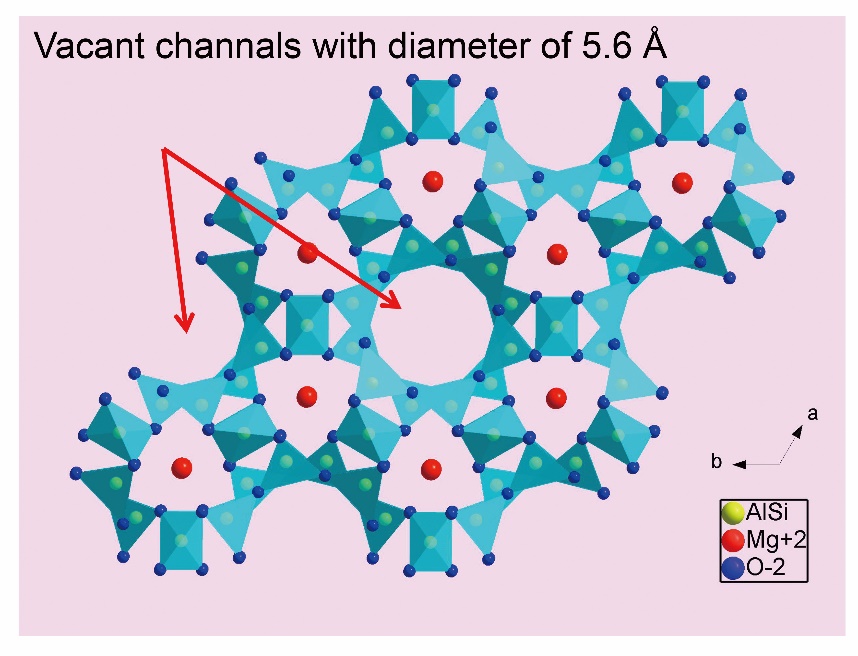


**Fig. S6:** Crystal structure of hexagonal Mg_2_Al_4_Si_5_O_18_, viewing along *c*-axis.


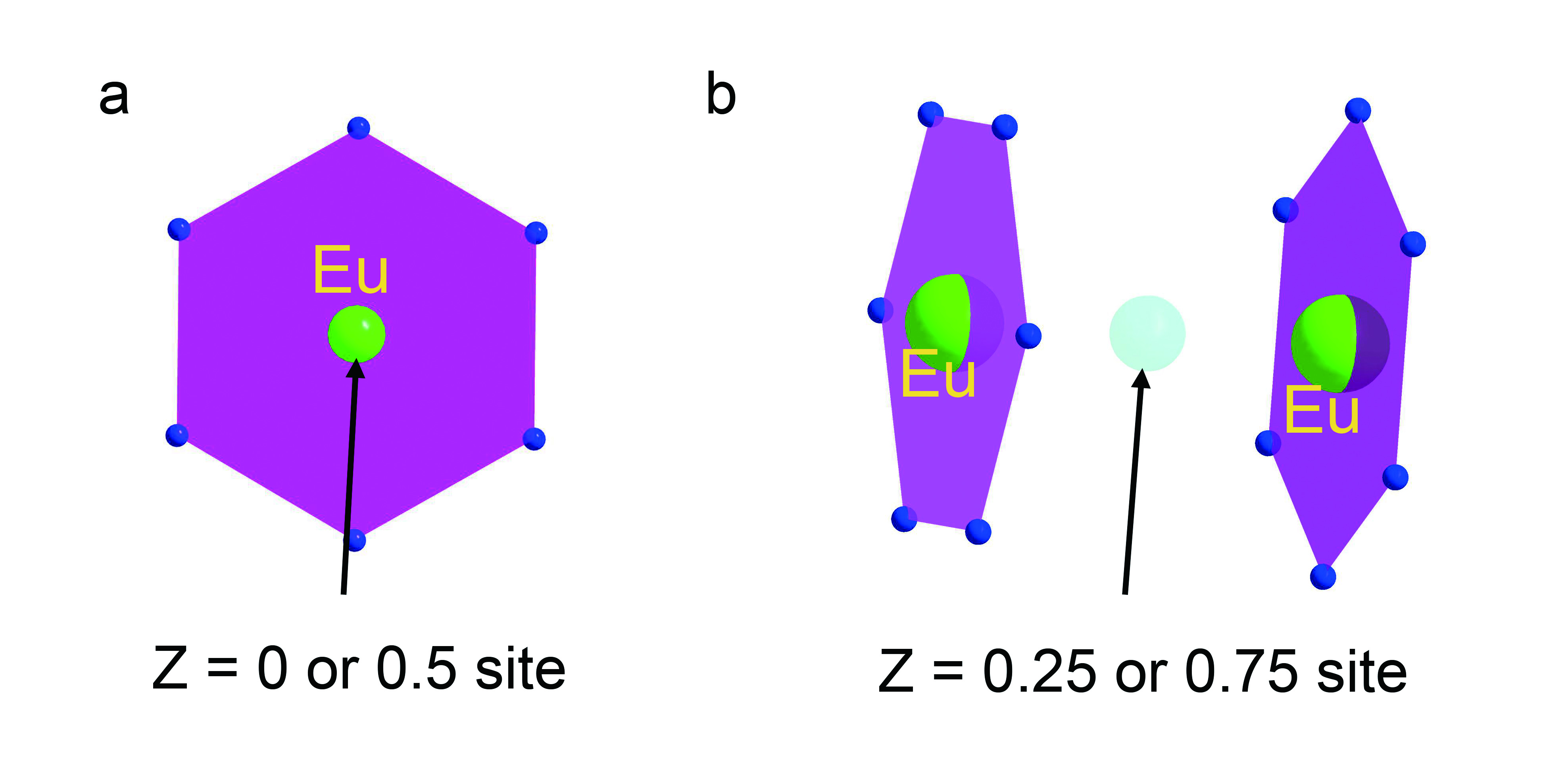


**Fig. S7:** Two different channel sites. **a** one is at z=0 or 0.5 with six-fold coordinated planar oxygen. **b** another is at z = 0.25 or 0.75 position coordinated with twelve oxygen.


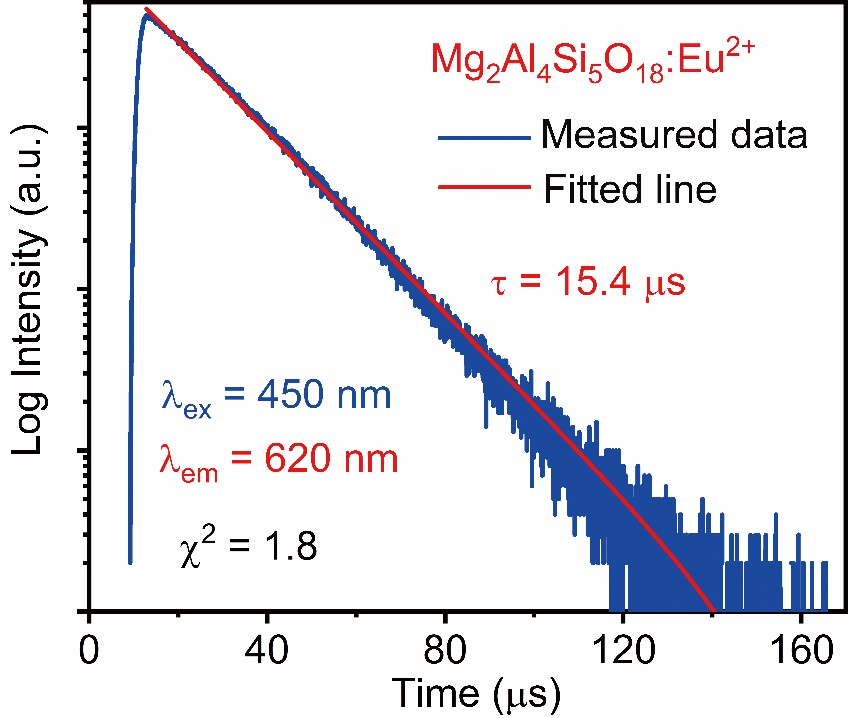


**Fig. S8:** PL decay (λ_ex_ = 450 nm, λ_em_ = 620 nm) of the crystallized Mg_2_Al_4_Si_5_O_18_: Eu^2+^ composite.


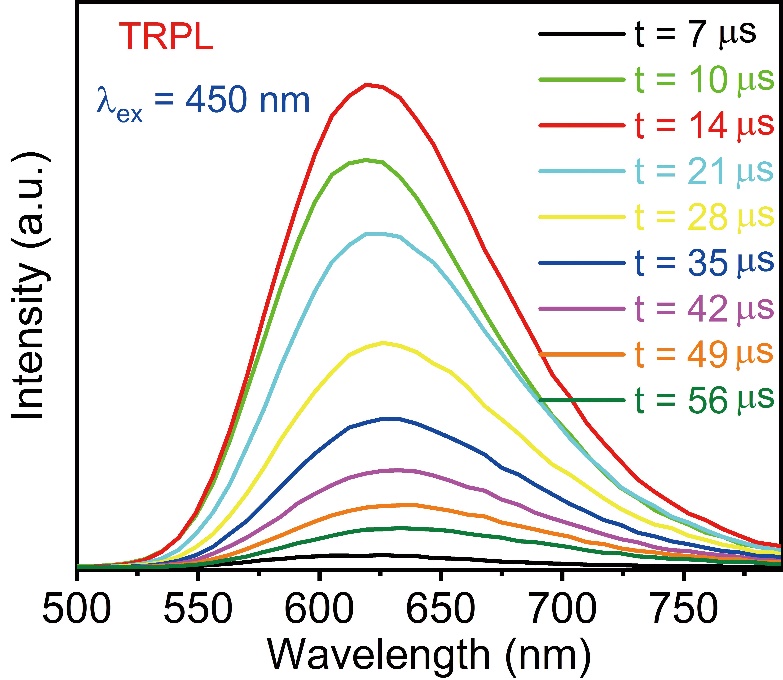


**Fig. S9:** Time-resolved emission spectra of the glass crystallized red Mg_2_Al_4_Si_5_O_18_:Eu^2+^ measured under excitation of 450nm pulse light. The spectra show an insignificant variation of emission profile with only a slightly shifts of emission towards lower energy as time prolongs from 7 to 56μs, suggesting that chemical surroundings around Eu^2+^ seems nearly homogeneous and the slightly shifts of emission energy is probably caused by the highly disordered Al/Si atoms in hexagonal Mg_2_Al_4_Si_5_O_18_ crystal.


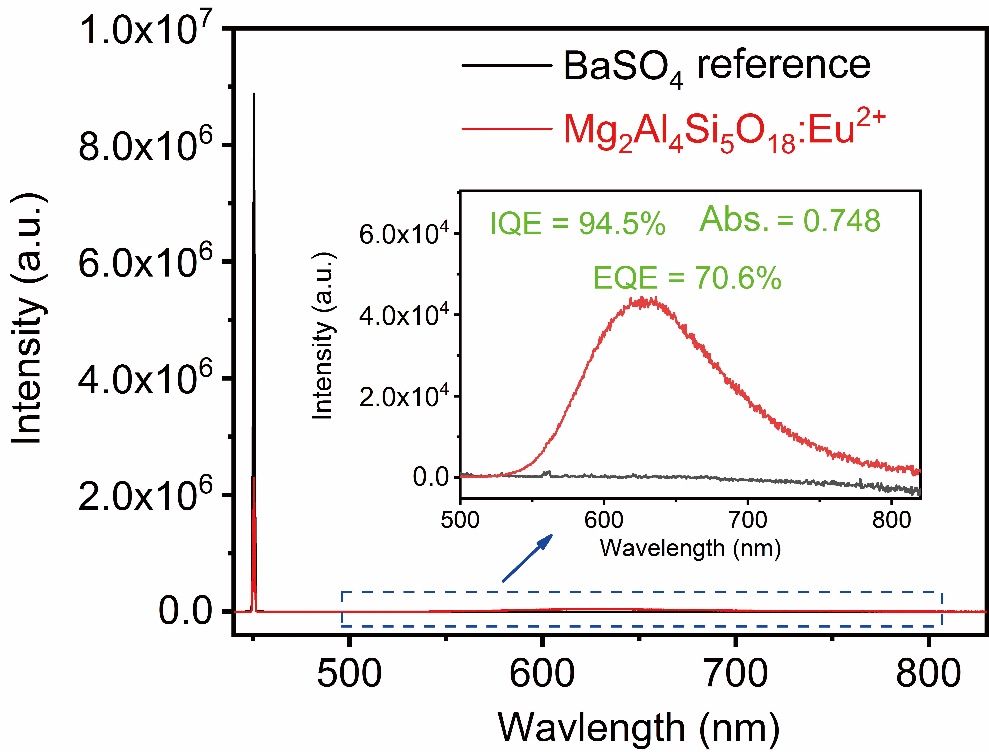


**Fig. S10:** Quantum emission efficiency of the glass crystallized Mg_2_Al_4_Si_5_O_18_:Eu^2+^ composite.


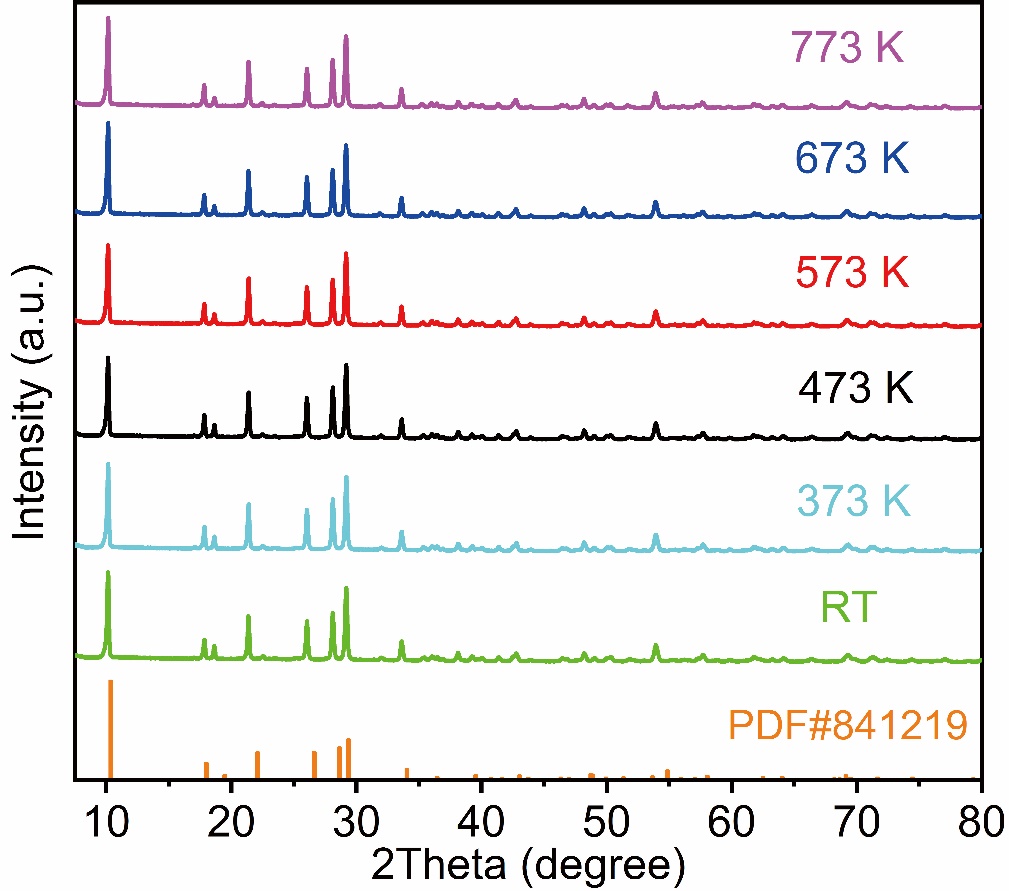


**Fig. S11:** Temperature-dependent XRD patterns of the crystallized Mg_2_Al_4_Si_5_O_18_: Eu^2+^.


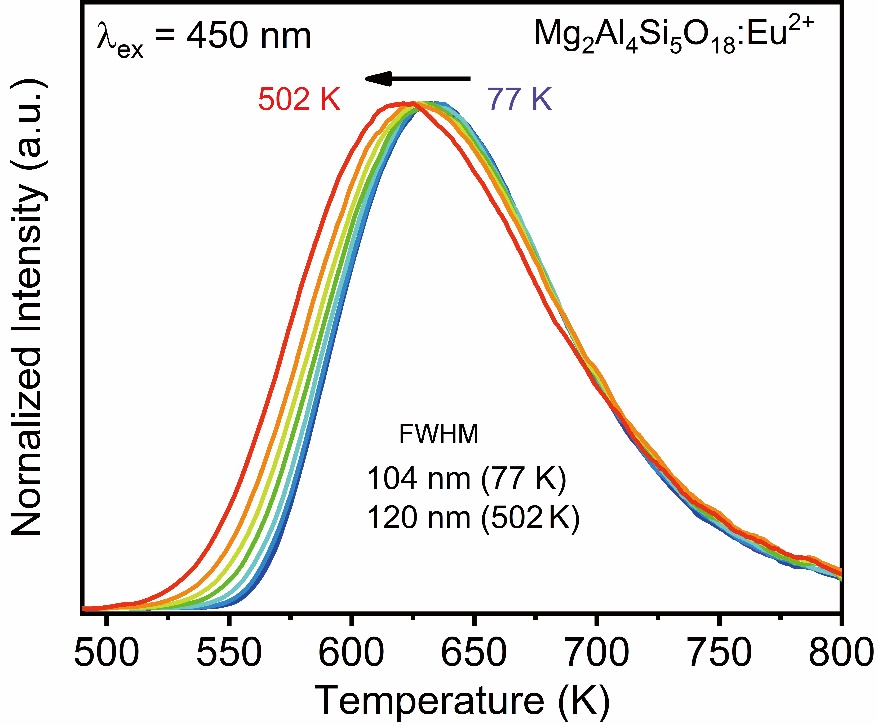


**Fig. S12:** Normalized Temperature-dependent PL spectra the Mg_2_Al_4_Si_5_O_18_: Eu^2+^ in the temperature range of 77 -502 K.


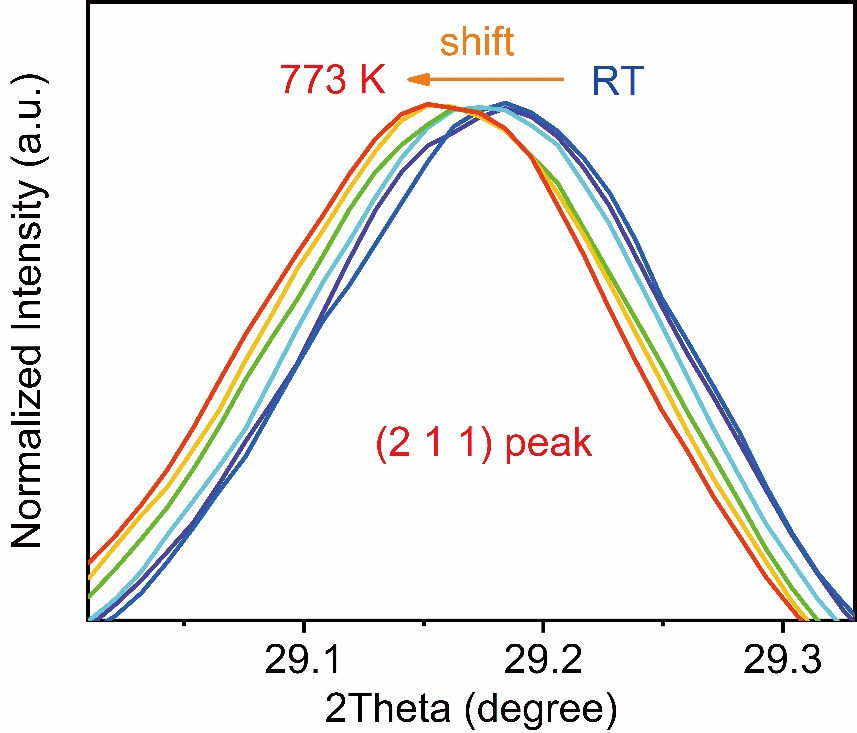


**Fig. S13:** Normalized temperature-dependent XRD patterns of the Mg_2_Al_4_Si_5_O_18_: Eu^2+^ showing the (211) diffraction peaks in the temperature of room temperature to 773 K.


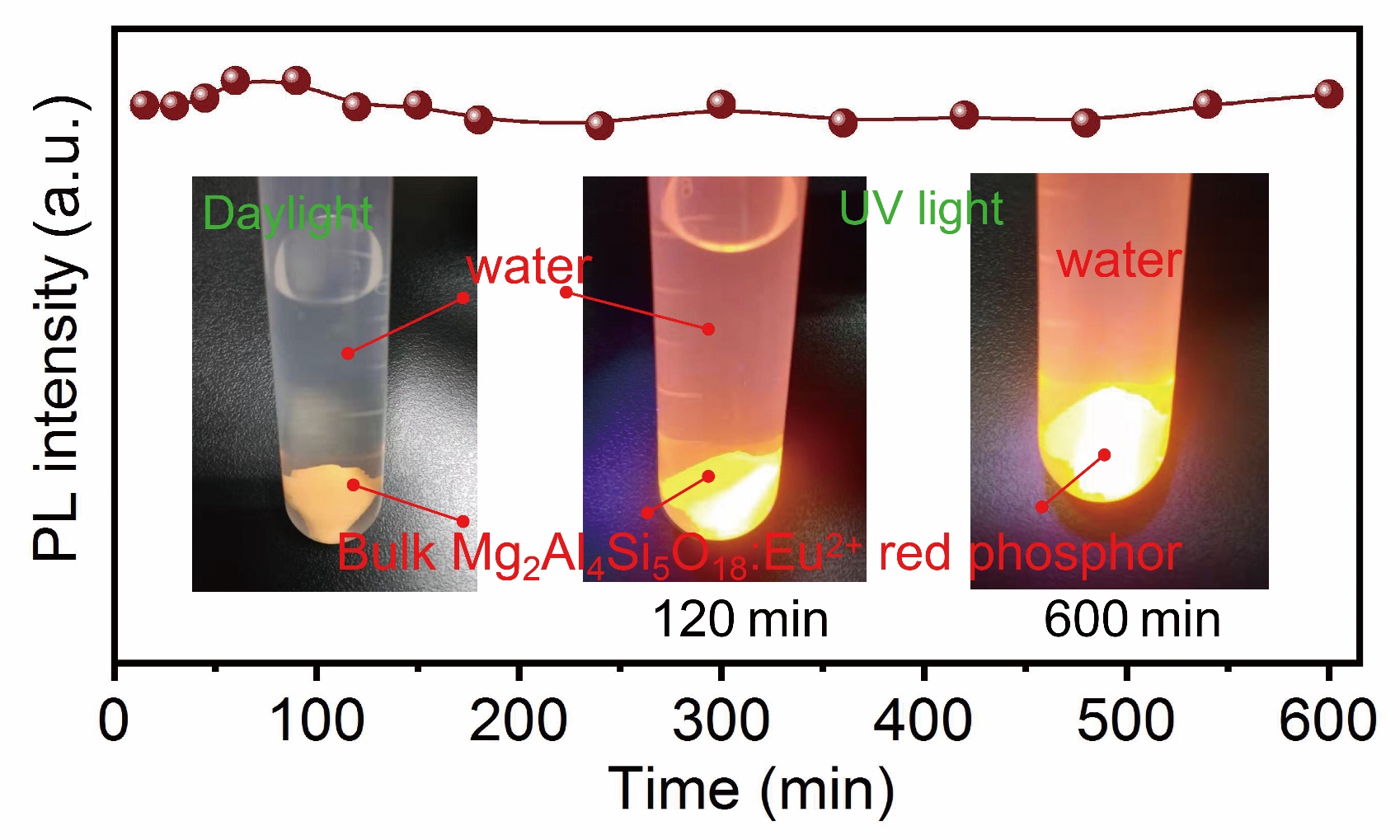


**Fig. S14:** Anti-moisture test by directly immersing the bulk Mg_2_Al_4_Si_5_O_18_:Eu^2+^ in water for 600 min. The inset shows the photographs of the corresponding luminescent photographs taken under daylight and UV light irradiation. The luminescence keeps at a constant level implying the good moisture resistance properties of the phosphor.


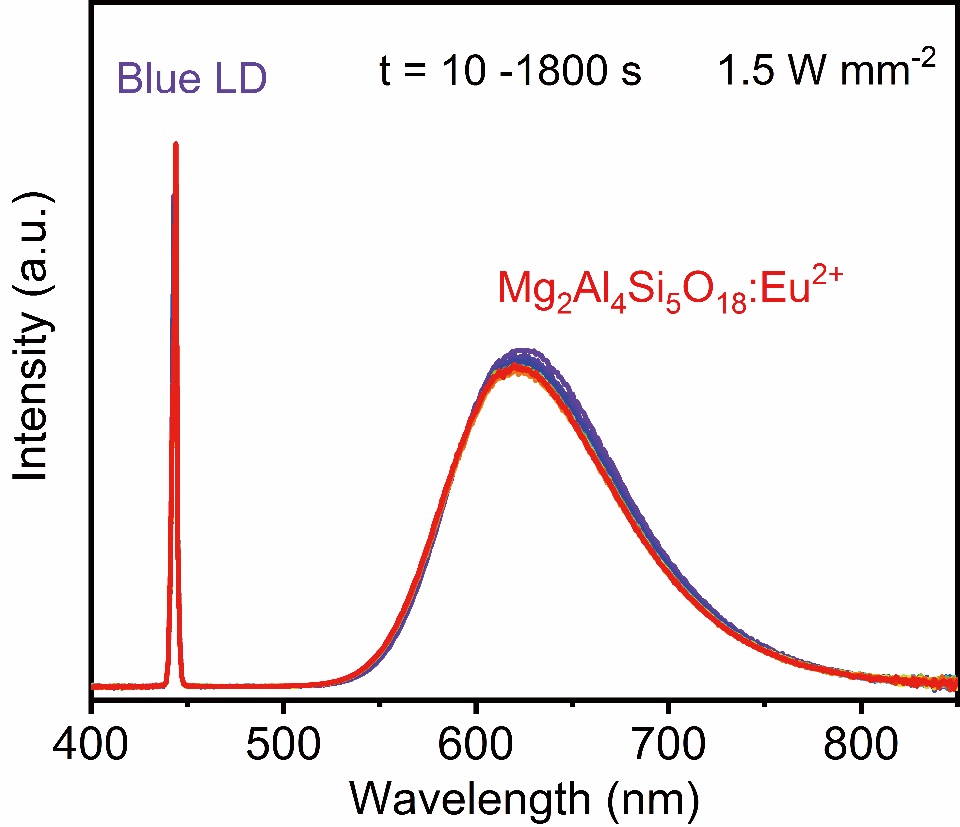


**Fig. S15:** Time-dependent emission spectra of the composite under a fixed incident laser power density of 1.5 W mm^-2^.

**Table S1.** ESD results (atom%) of the PG

|  | Mg | Al | Si |
| --- | --- | --- | --- |
| Atom ratio (%) | 10.21 | 11.17 | 15.24 |

**Table S2**. Main parameters of processing and refinement of the Mg_2_Al_4_Si_5_O_18_:Eu

| Compound | Mg_2_Al_4_Si_5_O_18_:Eu |
| --- | --- |
| Sp.Gr. | *P*6/*mcc* |
| *a*, Å | 9.7863 (3) |
| *c*, Å | 9.3718 (3) |
| *V*, Å^3^ | 777.31 (5) |
| *Z* | 2 |
| *2θ*-interval, º | 3-123 |
| *R_wp_*, % | 4.41 |
| *R_p_*, % | 2.90 |
| *R_exp_*, % | 0.57 |
| *χ^2^* | 7.71 |
| *R_B_*, % | 2.21 |

**Table S3.** Fractional atomic coordinates and isotropic displacement parameters (Å^2^) of Mg_2_Al_4_Si_5_O_18_:Eu

|  | *x* | *y* | *z* | *B*_iso_ | *Occ.* |
| --- | --- | --- | --- | --- | --- |
| Mg | 1/3 | 2/3 | 1/4 | 0.767 (19) | 1 |
| Si1 | 1/2 | 1/2 | 1/4 | 0.423 (15) | 0.09 (3) |
| Al1 | 1/2 | 1/2 | 1/4 | 0.423 (15) | 0.91 (3) |
| Si2 | 0.37216 (5) | 0.26597 (5) | 0 | 0.725 (12) | 0.77 (2) |
| Al2 | 0.37216 (5) | 0.26597 (5) | 0 | 0.725 (12) | 0.23 (2) |
| O1 | 0.48403 (7) | 0.34898 (7) | 0.14445 (6) | 0.724 (16) | 1 |
| O2 | 0.22775 (11) | 0.30539 (9) | 0 | 1.49 (2) | 1 |

**Table S4.** Main bond lengths (Å) of Mg_2_Al_4_Si_5_O_18_:Eu

| Mg—O1^i^ | 2.1143 (6) | Si2—O2^ii^ | 1.6185 (9) |
| --- | --- | --- | --- |
| Si1—O1 | 1.7194 (6) | Al2—O1 | 1.6739 (6) |
| Al1—O1 | 1.7194 (6) | Al2—O2 | 1.6405 (7) |
| Si2—O1 | 1.6739 (6) | Al2—O2^ii^ | 1.6185 (9) |
| Si2—O2 | 1.6405 (7) |  |  |

Symmetry codes: (i) -*x*+1, -*y*+1, *z*; (ii) *y*, -*x*+*y*, -*z*

**Table S5.** EXAFS fitted structure parameters at the Eu *L*_3_-edge（*Ѕ*_0_^2^=0.99）

| Sample | Path | C.N. | R (Å) | σ^2^×10^3^ (Å^2^) | ΔE (eV) | R factor |
| --- | --- | --- | --- | --- | --- | --- |
| Mg_2_Al_4_Si_5_O_18_:Eu^2+^ | Eu-O | 6.5±1.6 | 2.60±0.03 | 20.4±4.4 | 6.0±1.5 | 0.006 |
|  | Eu-Al | 9.0±2.1 | 3.33±0.02 | 11.5±2.5 | 11.8±1.7 |  |

*^a^N*: coordination numbers; *^b^R*: bond distance; *^c^σ*^2^: Debye-Waller factors; *^d^* Δ*E*_0_: the inner potential correction. *R* factor: goodness of fit. * the experimental EXAFS fit of metal foil by fixing CN as the known crystallographic value.

**Table S6.** Some important photoelectric parameters of the white LD driven at various incident power density.

| Power density  /Wmm^-2^ | CIE coordinate | CRI(R_a_) | CCT/K | LF/lm | LE/lmW^-1^ |
| --- | --- | --- | --- | --- | --- |
| 0.25 | (0.364, 0.333) | 85.2 | 4146 | 47.06 | 94.12 |
| 0.5 | (0.361, 0.333) | 83.7 | 4288 | 84.87 | 87.87 |
| 0.75 | (0.357, 0.333) | 82.2 | 4434 | 123.9 | 82.6 |
| 1.0 | (0.354, 0.333) | 80.8 | 4556 | 160 | 80.01 |
| 1.25 | (0.351, 0.333) | 79.4 | 4681 | 192.5 | 79.5 |
| 1.5 | (0.346, 0.333) | 77.4 | 4888 | 242.7 | 80.90 |
| 1.75 | (0.341, 0.333) | 75.3 | 5128 | 274.26 | 78.36 |
| 2.0 | (0.336, 0.333) | 73.8 | 5349 | 302.5 | 75.63 |
| 2.25 | (0.332, 0.332) | 72.6 | 5531 | 333.9 | 74.2 |
| 2.5 | (0.327, 0.331) | 71.3 | 5748 | 373.6 | 74.7 |
| 2.75 | (0.325, 0.330) | 70.6 | 5866 | 395.3 | 71.8 |
| 3.0 | (0.322, 0.327) | 70.0 | 6011 | 417.48 | 69.58 |
| 3.25 | (0.318, 0.3235) | 69.4 | 6226 | 440.37 | 67.75 |
| 3.5 | (0.314, 0.319) | 68.7 | 6525 | 462 | 66 |
| 3.75 | (0.307, 0.310) | 68.1 | 7049 | 477.8 | 63.7 |
| 4.0 | (0.305, 0.307) | 67.9 | 7255 | 480 | 60 |
